# Unravelling functional neurology: a scoping review of theories and clinical applications in a context of chiropractic manual therapy

**DOI:** 10.1186/s12998-017-0151-1

**Published:** 2017-07-18

**Authors:** Anne-Laure Meyer, Amanda Meyer, Sarah Etherington, Charlotte Leboeuf-Yde

**Affiliations:** 1 Complexité, Innovation et Activités Motrices et Sportives, Université Paris-Saclay, 91405 Orsay Cedex, France; 2 Complexité, Innovation et Activités Motrices et Sportives, Université d’Orléans, 45067 Orléans, France; 3 Institut Franco Européen de Chiropraxie, 24 Bld Paul Vaillant Couturier, 94200 Ivry sur Seine, France; 4 School of Health Professions, Murdoch University, 90 South Street, Murdoch, W.A 6150 Australia; 5 School of Veterinary and Biomedical Sciences, Murdoch University, 90 South Street, Murdoch, W.A 6150 Australia

**Keywords:** Functional neurology, Chiropractic, Spinal manipulation, Scoping review

## Abstract

**Background:**

Functional Neurology (FN), a seemingly attractive treatment approach used by some chiropractors, proposes to have an effect on a multitude of conditions but some of its concepts are controversial.

**Objectives and design:**

A scoping review was performed to describe, in the context of chiropractic manual therapy, 1) the FN theories, and 2) its clinical applications (i.e. its indications, examination procedures, treatment modalities, treatment plans, and clinical outcomes) using four sources: i) one key textbook, ii) the scientific peer-reviewed literature, iii) websites from chiropractors using FN, and iv) semi-structured interviews of chiropractors using FN.

**Methods:**

The scientific literature was searched in PubMed, PsycINFO, and SPORTDiscus, completed by a hand search in the journal *Functional Neurology, Rehabilitation and Ergonomics* (November 2016 and March 2017, respectively). The only textbook on the topic we found was included and articles were chosen if they had an element of manual therapy. There was no restriction for study design but discussion papers were excluded. Websites were found in Google using the search term “Functional Neurology”. Chiropractors, known to use FN, were invited based on their geographical location. Theories were mainly uncovered in the textbook as were all aspects of the clinical applications except treatment plans. The other three sources were used for the five aspects of clinical applications. Results were summarized and reported extensively in tables.

**Results:**

Eleven articles were included, five websites scrutinized, and four semi-structured interviews performed. FN is based on the belief that reversible lesions in the nervous system are the cause of a multitude of conditions and that specific clusters of neurons can be positively affected by manipulative therapy, but also by many other stimuli. Diagnostic procedures include both conventional and unusual tests, with an interpretation specific to FN. Initial treatment is intense and clinical outcomes reported as positive.

**Conclusion:**

FN gives the impression to be a complex alternative to the old variant of the chiropractic subluxation model, in which the vertebral subluxation is replaced by “physiological lesions” of the brain, and the treatment, spinal adjustments, are complemented by various neurological stimuli. Both models purport to treat not the symptoms but the cause. We conclude there is a need for more scientific documentation on the validity of FN.

**Electronic supplementary material:**

The online version of this article (doi:10.1186/s12998-017-0151-1) contains supplementary material, which is available to authorized users.

## Background

Chiropractic is a health profession that is legally recognized in several countries under a regulatory framework to deal with neuromusculoskeletal conditions [1, 2]. Spinal manipulation is one of the key aspects of chiropractic treatment, often combined with advice on life-style, physical activities, specific exercises, and ergonomics [3, 4]. Clinical experience shows that manipulation of joints can have a pain-reducing effect, and this has also been confirmed in purely experimental studies [5], providing at least some evidence for the approach.

However, some chiropractors propose therapeutic solutions outside the recognized scope of chiropractic practice. For as long as chiropractors have existed, some practitioners have also treated non-musculoskeletal conditions such as asthma, colic in children, and dysmenorrhea, although this part of clinical practice has been less common than the treatment for musculoskeletal conditions [4, 6, 7]. The rationale for such treatment is that spinal manipulation can have an effect also on the autonomic nervous system [8].

There are several currents within chiropractic that purport to successfully treat various non-musculoskeletal conditions. One such approach is Functional Neurology (FN), which, at first glance has as its rationale the concept that disturbances of the physiology in the nervous system, especially those in the brain, can have many detrimental effects on the body. These disturbances are proposed to be often reversible or at least to have the potential for improvement. The list of conditions proposed to be successfully treated by FN is extensive, the diagnostic procedures complex and the therapeutic approach often multi-facetted.

When attempting to review the origins of FN, the earliest reference to FN found in the scientific literature is an article from 1997 by a chiropractor, also presented as the founder of FN, FR Carrick [9]. In this article, he stated that spinal manipulation can alter the size of the physiological blind spot of the eye in certain cases, a phenomenon that he claimed was a proof that spinal manipulation has an effect on brain function. In that article, there is also a lengthy presentation of the presumed causative link between spinal manipulation and brain function. This work elicited several responses to the editor, with many questions and criticisms [10–16], but also generated positive comments [17–19].

Despite the criticism that FN has encountered over the last 20 years, both inside and outside the profession [10, 14–16, 20, 21], FN appears to have become an attractive discipline for many chiropractors [3, 22], promoted by some as a discipline at the cutting edge of science [22, 23]. For example, a recent survey of the Australian chiropractic workforce reports that 13.3% of the respondents use FN [3]. Other health practitioners (e.g. medical doctors, physiotherapists) also seem to be interested in this new discipline, having access to FN courses [24].

The main criticism leveled at FN concerns the lack of scientific basis [10, 14–16, 20, 21]. In addition, published information seems to be sparse and, for the uninitiated, the subject is complex. An overview of FN would therefore be of value to chiropractors, students and chiropractic educators, with an interest in FN to: 1) provide a basic description of its concepts and their clinical applications and 2) to present the scientific evidence underlying these basic concepts.

In this scoping review we will focus on the first point by attempting to provide a basic description of FN concepts and their clinical applications, in the context of chiropractic manual therapy. Our six research objectives were to describe: 1) the theories that constitute the basis of FN, 2) the conditions that functional neurologists treat, 3) the diagnostic procedures, 4) the therapeutic modalities, 5) the course of care, and 6) the clinical outcomes obtained or expected with this approach.

The field of FN is large, composed of different sub-specialties [25], some of which have developed somewhat different directions than the original one. The work of G Leisman and R Melillo in the area of FN applied to childhood neurodevelopmental disorders is an example of such sub-specialties [26]. Nevertheless, it appears to be practiced primarily by chiropractors. In fact, FN is also known as “Chiropractic Neurology”. For these reasons, we have limited our review of the literature to the fundamental concepts of FN and/or with FN as a supplement to “traditional” chiropractic, i.e. which would typically include the use of manual therapy.

## Method

### Design and brief description of study

In order to obtain information on our six research objectives, we performed a scoping review using three written sources and one semi-structured interview, as briefly described below. Scoping reviews are often used to obtain a preliminary understanding of a poorly understood topic, have a non-rigid but systematic approach, allow for multiple methods, and do not necessitate a critical element [27, 28]. Although there are currently no strict methodological rules for conducting scoping reviews, we endeavored to follow the six steps of the Arksey and O’Malley framework [27].

Initially, the first author read the only comprehensive textbook on the concepts of FN that was found [29]. We used this source as the basis or starting point for our future work in order to gain an understanding of the FN theoretical background. Thereafter, we consulted the scientific literature to see what information was available and searched the internet to obtain an idea of how practitioners, who state that they practice FN, describe their activities. Also, we interviewed a number of practitioners who use FN in their daily practice, making it possible to ask clarifying questions. To allow for ease of reading, several aspects of the various methods have been described in Additional files 1, 2 and 3.

### Search strategy for information

#### Written information

##### Textbook

We had access to a textbook [29] that served as our first source of information. This book, recommended by organizations such as the American Chiropractic Neurology Board and the Functional Neurology Society, is authored by a chiropractor, RW Beck, with the foreword written by FR Carrick.

##### Scientific literature

It was difficult to find scientific literature on FN using the usual search strategies, for which reason alternative methods were employed. These have been described in Additional file 1. Briefly, a search by name of author on PubMed, PsycINFO, and SPORTDiscus was conducted. This was complemented by contacting by email a number of practitioners and/or researchers known to be involved in FN to ask them for their updated publication list. Following this step, we searched for articles in the journal *Functional Neurology*, *Rehabilitation, and Ergonomics*, which has FN among its aims and scope. This journal was recommended by one of the researchers involved in FN.

##### Websites

The internet was searched via Google using the keyword “Functional Neurology” in order to capture a number of professional websites of chiropractors presenting themselves as functional neurologists.

#### Interviews

Through our network of contacts, we identified European-based chiropractors who used FN in their daily practice and who were likely to participate in a future interview. A convenience sample consisting of five of those, all living in France, where also the chief investigator was located, were finally invited. The four who replied were interviewed. These four chiropractors were contacted by email, provided with information about the survey and asked to provide informed consent.

### Inclusion and exclusion criteria of articles and websites

#### Scientific articles

Articles were included if they described studies on a FN therapeutic approach to one or more specific condition(s) or if they described studies on a FN therapeutic approach on healthy or non-healthy subjects with positive clinical sign(s). Also, the articles had to include the use of manual therapy. Articles written by functional neurologists dealing with issues such as medication use or modified states of consciousness were not included. Discussion papers, abstracts, poster presentations, conference papers, and letters to the editors were excluded.

#### Websites

Websites of chiropractors describing themselves as functional neurologists were included if they clearly mentioned that they were *Diplomates of the American Chiropractic Neurology Board* (DACNB), as this seems to indicate that the person has obtained a certain level of proficiency on this topic. There were no restriction criteria regarding their nationality or their number of years of experience in FN. However, the search was restricted to websites written in English.

### Inclusion criteria for the interview

Our inclusion criteria were that the chiropractors were DACNB or, at least, in active training. They also had to be willing to clarify the basic concepts of FN and to describe the applications of FN in their daily practice during a semi-structured interview. We selected the four French chiropractors for geographical reasons, as the research team was located in Paris.

### Collection of relevant information

#### Written information

##### Textbook

The chapters of interest of the textbook were selected based on its table of contents. The whole book was read prior to this in order to attempt to gain a good understanding of the topic.

##### Scientific articles

The first investigator searched the databases and publication lists forwarded by the authors on request (see Additional file 1) and selected the potentially relevant full texts from titles and abstracts. As the authors were not familiar with FN, the selection of potentially relevant full texts was generous. ALM and CLY independently made the search in the journal *Functional Neurology*, *Rehabilitation, and Ergonomics* and selected the potential relevant full texts from titles and abstracts. All full texts were independently assessed in relation to the inclusion and exclusion criteria. In addition, the first investigator searched reference lists for relevant articles from the databases and the journal.

##### Websites

Once the first author had found the mention that the chiropractors were DACNB, the corresponding websites were screened (except for their blog section) sequentially, in the order by which they appeared in a Google search conducted in September 2016. This was performed by searching for terms in relation to FN and our research objectives. When no new information was found for one topic, search was stopped for this topic but continued for the others until no new information was found. Texts were documented with screenshots.

#### Interviews

There are no strict rules for how to conduct or interpret interviews in scoping reviews. Relevant information was collected through a semi-structured interview designed by the first author and another PhD student. It was tested on one of the chiropractors, after which some improvements were made, mainly to the wording of the questions. The interview contained twenty-four questions, eleven were used in this review and the others will be used elsewhere (see Additional file 1). Clarifying questions were added as needed during the interviews. The interview instrument was constructed based on our specific questions related to FN and thus had not been previously used, tested or validated.

#### Ethical considerations

According to French law [30, 31], no ethics permission is required when interviewing consenting adults in a non-interventional context. However, the written consent of each interviewed chiropractor concerning the recording of the interview and its use as research material was obtained. Furthermore, no personal information was collected and all results were reported anonymously. The transcribed versions were provided to the interviewees for comments.

### Extraction of information

#### Written information

The information from the textbook by RW Beck [29] was retrieved by the first author from specific chapters almost entirely dedicated to our topics of interest. Chapters 1, 4 and 18 were used to extract the theories, which were complemented by information from chapters 3, 9, 19 and 20. Chapter 19 and, to a lesser extent chapter 20, were used to extract the indications. Chapter 4 and, to a lesser extent chapter 19, were used for examination procedures. Chapter 20 and, to a lesser extent chapter 19, were used for treatment modalities. The information related to the outcomes of treatment were extracted from chapter 19. Despite a chapter dedicated to clinical cases, there was no detailed information on treatment plans. References in this text (section “Neurophysiological theories”) refer directly to these chapters and relevant pages to assist the reader who might want to compare our information with that of the textbook.

Descriptive checklists were created to collect systematic information from the scientific articles and from the websites, in relation to the research objectives (available in Additional file 2a and b). The format and contents were somewhat different, depending on the data source. For example, the websites were expected to provide information on expected outcome rather than reported outcome whereas the reverse was expected from the scientific literature. The search for relevant information in the scientific literature was done independently by two authors (ALM and CLY). The descriptive checklist for the websites was completed by the first author who blindly performed this procedure twice for each website.

#### Interviews

Each interview was taped and transcribed in a narrative form and in a tabulated form to better visualize the information (tabulated form is available in Additional file 2c). They were conducted by the first author and another PhD student, one of whom was responsible for the narrative transcript of two of the interviews and the other for the transcript of the remaining two. After agreement between the two interviewers on the content of each narrative transcription (tapes were available in case of disagreement), the transcript was sent to the interviewed chiropractor to obtain his/her agreement on its content. Absence of feedback was interpreted as an acceptance of the text (the interviewees were informed of this). Thereafter, based on each narrative transcript, the two interviewers independently extracted and collated information by themes in a table, which was created in relation to the research objectives of the review. The content of their respective tabulated transcript was compared for agreement (final table is available in Additional file 2c).

### Data analysis and synthesis

Initially, the first author identified which of the four sources had dealt with the various research objectives (see Table 1). Thereafter, we concentrated on one item at a time, collecting the relevant information either in a table or as narrative text. The multiple methods are detailed in Additional file 3. A narrative synthesis was done for each research objective, based mainly on the tabulated overview of the information.

**Table 1:** Sources used in a scoping review on Functional Neurology to obtain information on six research objectives

Research objectives	Book (*n* = 1)	Scientific articles		Websites (*n* = 5)	Interviews (*n* = 4)
		Randomized controlled trial and controlled trial (*n* = 2)	Case reports (*n* = 9)		
Theories	1				
Indications	1		9	5	4
Diagnostic procedures	1	2	9	4	4
Therapeutic modalities	1	2	9	5	4
Treatment plans			7	3	3
Clinical outcomes	1		9	5	4

## Results

### General information

#### Textbook

The textbook provided information for five of our objectives (theories, indications, diagnostic procedures, treatment modalities and short-term outcomes). As shown in Table 1, it was the only source that could clearly be used to describe the theories of FN. We selected some of the major concepts of FN, which we have attempted to describe in the text below (“Detailed results”) in order to capture the theoretical framework of FN, as presented in this book.

#### Scientific articles

**Fig. 1:**
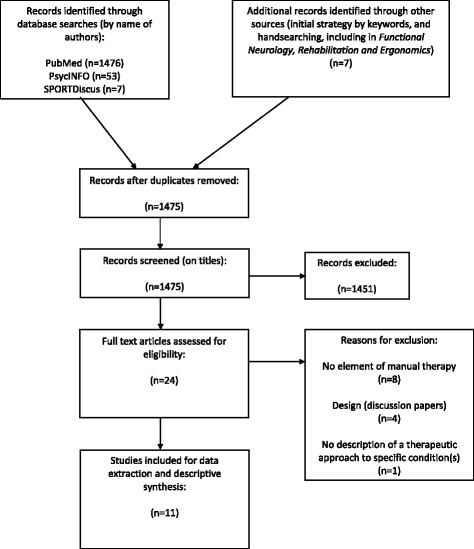
Description of the search for literature in a scoping review of Functional Neurology in a chiropractic context

The selection process is summarized in Fig. 1. Three case reports, one controlled trial, and one randomized controlled trial were found in our areas of inquiry in the selected databases. Six case reports were found through hand searching, including three in the journal *Functional Neurology*, *Rehabilitation, and Ergonomics*. Lists of publications obtained from known FN researchers and/or practitioners did not provide any additional relevant material. Nevertheless, the scientific literature provided information on diagnostic procedures, treatment modalities, treatment plans and clinical outcomes for various conditions. The controlled trial and the randomized controlled trial provided information only on diagnostic procedures and treatment modalities (see Additional file 3 for details). Moreover, two case studies did not report the treatment plan and, another case study did not report the brain areas targeted by the treatment. There was no disagreement between the two authors who independently collected the information from the scientific literature.

#### Websites

The search for information on the internet was saturated fairly quickly. We did not find any new information after reading the full content of the first five websites on Google. These all belonged to chiropractors practicing in the United States. The internet search provided particular information on the indications, diagnostic procedures, and treatment plans. Only one website provided information on treatment in relation to a specifically targeted area in the brain. Furthermore, the websites generally gave only general information about the expected outcomes following FN treatment. One website did not provide information about the diagnostic tests used by the chiropractors and two websites did not provide relevant information related to treatment plans except to say that they were individualized.

#### Interviews

The first interview lacked somewhat in clarity and thus served as a pilot interview. However, the following three interviews provided clear and extensive answers to our questions. All four interviews could be used in this study as they provided fairly homogeneous information. Therefore it was deemed unnecessary to collect data from additional practitioners. The transcripts sent to the participants needed only few minor revisions on their part; three of them gave us feedback. The interviews informed us mainly about indications, diagnostic tests, and treatment modalities. We were able to collect less information related to treatment plans (3/4 informants) and expected outcomes, for which only general information was provided. The tabulated transcriptions (available in Additional file 2c) made by the first author and the other PhD student revealed no obvious difference in their content.

In the section below, the theories of FN are reported first in relation to the neurophysiology, thereafter in terms of treatment implementations. This work aims to provide a basic description of these theories and does not pretend to cover them in depth. Although we attempted to report these theories of FN faithfully, the text below represents our understanding of FN derived from this study, which does not necessarily depict the official view of FN. Finally, the five different aspects of the clinical applications of FN are discussed, based on our four sources of information.

### Detailed results

#### Neurophysiological theories: (*information from textbook*)

The practice of FN includes the detection, evaluation and conservative treatment of functional aberrations of the neuraxis, especially of the brain [32]. Within FN, neurological aberrations are named “physiological lesions” or “functional lesions”. They are stated to be the cause of a large number of unlabeled, poorly labeled or misunderstood symptoms in the medical field (e.g. neurodevelopmental disorders, movement disorders) [33]. By the same reasoning, FN proposes explanations also for musculoskeletal disorders.

These “physiological lesions” are described as reversible due to the neuroplastic properties of the nervous system and may affect any parts of the nervous system. “Physiological lesions” are different from “ablative lesions” that are defined as only potentially and very slowly reversible, as they have resulted from death of neuronal tissues (e.g. post-stroke). These two types of lesions would lead to very similar symptoms and could coexist [34].

The textbook information on the neurophysiological rationale of FN can be broadly classified under three headings:

1. Cellular level,
2. Related neurological pathways,
3. The FN concept of “hemisphericity”.

For each of these, we found:

1. A description and interpretation of core neurophysiological and/or neuroanatomical information,
2. A description of consequences when the neurophysiology goes wrong (possible disorders and symptoms), which, may or may not represent the generally acceptable view in the scientific world,
3. Methods to test the integrity of various groups of neurons, most often indirectly, especially those located in the brain.

Some of the major components of the theoretical framework of FN will be reported following the above classification.

#### Cellular level: (*information from textbook*)

At a cellular level, the central tenet of FN is that symptoms result from a dysfunctional “central integrative state” (CIS) of one or several functional units of neurons within the nervous system (e.g. group of neurons of the right dentate nucleus). In other words, a “physiological lesion” corresponds to a group of neurons with a dysfunctional CIS. More precisely, such lesions would occur following disturbances of neuronal physiology that in turn would affect communication within the central nervous system, leading to hyper and/or hypo-functional area(s) within certain areas of the brain. In response to this altered function, the concerned area(s) would send an abnormal quantity of outputs, i.e. too many or not enough, causing diverse motor, sensory, visceral or cognitive symptoms such as the ones listed in Table 2 and discussed later in this text [34]. In general, a “physiological lesion” is said to affect only one side of a brain structure (e.g. one side of the cerebellum or one cortical hemisphere), leading to asymmetries of outputs (aspect discussed later in section “The FN concept of “hemisphericity””).

**Table 2:** Indications for treatment using Functional Neurology according to four sources in a scoping review

Groups and/or subgroups of conditions		Source of information			
		Book	Articles	Websites	Interviews
Neuromusculoskeletal disorders	Headaches	NM	NM	X	X
	Others	Low back pain with radiculopathy Peripheral neuropathies	Low back pain Neck pain Ankle pain	Low back pain Radiculopathies Neck pain Peripheral neuropathies Spinal stenosis Upper/lower extremity conditions	Low back pain Radiculopathies Neck pain
Traumatic brain injuries (symptom(s) related to such injuries)		X	X	X	X
Neurological diseases or disorders	Neurodegenerative disease	Parkinson’s disease	Parkinson’s disease	Parkinson’s disease Multiple sclerosis Alzheimer’s disease	Parkinson’s disease Multiple sclerosis
	Movement disorders	Dystonias	Cervical dystonia	Dystonia Tremor disorders	Dystonia
	Post-stroke symptoms	X	NM	X	X
	Others	Migraines Complex regional pain syndrome Dysautonomia	Migraines Complex regional pain syndrome Landau Kleffner syndrome	Migraines Seizure disorders Spinal cord lesions Fibromyalgia Restless legs	Migraines
Psychiatric disorders	Neurodevelopmental disorders	ADHD	ADHD	ADD/ADHD Dyslexia Autism	ADD/ADHD « dys » disorders, including dyslexia
	Mood disorders	Anxiety Depression	NM^a^	Anxiety disorders Depression	NM
	Others	OCD	OCD Tourette’s syndrome	OCD PTSD	PTSD
Various neurological and non-neurological symptoms		Tinnitus Deafness Muscle spasms Post manipulative therapy symptoms	Paresthesia	Balance disorders Vertigo Numbness Sleeping difficulties	Balance disorders Vertigo
Others		Oral dysplasia	Primary nocturnal enuresis	Physical, cognitive, academic and/or creativity enhancement Lyme disease	NM

*NM* Condition(s) not mentioned

*X* Condition(s) mentioned without specific example(s)

*ADD/ADHD* Attention deficit disorder / attention deficit and hyperactivity disorder

*OCD* Obsessive compulsive disorder

*PTSD* Post-traumatic stress disorder

^a^One reviewed article deals with mood disorders in a context of multiple symptoms related to traumatic brain injury

The CIS of a functional group of neurons appears to be considered as the “state of health” of those neurons. This state is said to be determined by three parameters that allow the survival and function of neurons: i) oxygen supply, ii) nutritional supply, and iii) stimulation, i.e. synaptic activation [34]. These three parameters have to be in an adequate amount to ensure a “healthy” CIS. Many factors, mainly external, would negatively modify the state of health of a functional group of neurons. An immobilization in a cast, an acute anoxic episode after attempting suicide, or an inappropriately performed spinal manipulation are examples of such proposed external factors [35, 36].

Evaluating the CIS of the different units of neurons of the central nervous system, especially those of the brain, is the central aim of the clinical examination within FN. As it cannot be performed directly, it is mainly evaluated through a detailed analysis of the responses of different effectors tested during the patient’s examination. These responses are proposed to be, to a large extent, determined by the CIS of the presynaptic neuronal pool(s) projecting to the neurons ending at the tested effector. A major sign of a dysfunctional CIS is described as a “fatigability” of the tested neurons, which means that the response to a continued or repeated stimulus cannot be sustained as it should [33]. An effector has to be tested bilaterally in order to find the faulty side, because of the concept of asymmetrical function of two parts of a brain structure. In addition, as a “physiological lesion” can result in symptoms qualified as “subclinical”, functional neurologists have to attempt to detect “minor” asymmetries. This concept, in FN that “minor” asymmetries are clinically relevant, makes up one of the big differences between FN and classical neurology [33].

#### Related neurological pathways: (*information from textbook*)

To assess the CIS of one or several neuronal units and to elaborate an individualized treatment plan, solid knowledge in neuroanatomy is needed, as a “physiological lesion” could occur at any point along a neural pathway. Some pathways are identified as being of particular importance to a FN assessment, such as the cortico-reticulo-spinal tract that is described as beginning at a cortical hemisphere, passing mainly through the ipsilateral pontomedullary reticular formation (PMRF) and terminating, for most of the fibers, in the ipsilateral spinal cord [34]. We will use this particular pathway as an example to illustrate FN reasoning.

For functional neurologists, the importance of this pathway would relate to its following roles:

- Ipsilateral facilitation of muscle tone,
- Ipsilateral inhibition of anterior muscles above the spinal level of T6 and of posterior muscles below T6,
- Ipsilateral inhibition of pain sensation,
- Ipsilateral inhibition of sympathetic nervous system.

These functions are described as the result of the normal activation of the PMRF by the ipsilateral cerebral cortex.

In fact, this is a key pathway in FN, said to become disturbed if a “physiological lesion” on one side of the brain, in FN named “hemisphericity”, decreases the PMRF outputs. This decrease is described to be due to the decrease of the cortical outputs to the PMRF. Clinically, this would lead to:

- A global ipsilateral decrease of muscle tone,
- A “flexor angulation” of the ipsilateral upper limb and an “extensor angulation” of the ipsilateral lower limb, a posture known in FN as “pyramidal paresis”,
- One or more ipsilateral pain syndrome(s),
- An ipsilateral increase of sympathetic activity leading to a number of autonomic signs (e.g. increase of blood pressure, increased sweating, large pupil size) [34].

Combined, these clinical signs indicate that the patient would suffer from a “hemisphericity”, further discussed below.

#### The FN concept of “hemisphericity”: (*information from textbook*)

The concept of “hemisphericity” (also termed “cortical lateralization” or “brain asymmetry”) appears to be specific to FN, referring to a cerebral hemisphere suffering from a dysfunctional CIS. Thus, this is a “physiological lesion” that does not refer to a recognized pathological lesion such as a brain lesion caused by a stroke. Usually, it describes the side where the cortical activity is stated to be decreased. Within the FN framework, this concept rests on the assumptions that the two hemispheres: i) control different body functions, and ii) can function at two different levels of activation without there being an obvious pathology [34].

Widespread consequences are thought to result from this one-sided “physiological lesion”, including: cognitive (e.g. attention deficit disorder / attention deficit and hyperactivity disorder), psychiatric (e.g. depression), motor (e.g. muscle weakness), immune (e.g. systematic lupus erythematosus), and autonomic manifestations [32] (e.g. asymmetry of blood pressure). It is also considered that “hemisphericity” may lead to spinal manifestations and conditions such as: “subluxation”, modifications of the spinal curves, spondylosis, muscle stiffness, and muscles weakness of the intrinsic spinal muscles [34]. Such diagnoses, symptoms, or findings orientate the functional neurologist to the side of the dysfunctional hemisphere.

In addition to these clinical manifestations and to signs evoked above in relation to disturbance of the control of the PMRF outputs, other signs could be searched for and additional tests performed to diagnose a “hemisphericity”. Among them there are:

- Eye movement dysfunction(s),
- Contralateral cerebellar sign(s),
- Contralateral enlargement of the physiological blind spot of the eye, an, apparently, original concept of FN (see [9] or [33] for details about measurement of the physiological blind spot).

In fact, any neurological dysfunction that can be related somehow to aberrant cortical outputs is considered potentially relevant [37].

The concept of the physiological blind spot deserves some explanation because it belongs to the history of FN. In classical textbooks, the blind spot (optic disc) is described as the area of the retina devoid of photoreceptors, i.e. the area where converging retinal ganglion cells exit the eyeball to form the optic nerve. The perimeter of the blind spot can be mapped out during the examination of the visual field to detect some eye pathologies and to follow their progress [38]. However, FR Carrick [9] presents the claim that, in the absence of an eye pathology, the size of the blind spot can be altered in response to the CIS of the visual cortex and, by extension, the CIS of one hemisphere, which in turn would depend to a large extent of the afferent inputs it receives from the thalamus through the thalamocortical radiations. Related to this concept, spinal manipulation occupies a prominent place in FN, principally because of its proposed supra-segmental effects. Indeed, it is stated that spinal manipulation is able to generate changes in the size of the blind spot because of the afferent stimulation it would provide to the thalamus, in this way affecting the amount of afferent inputs to the cortex by the thalamus. Thus, manipulation is stated to have a direct effect on the brain, a central tenet within FN [32, 34]. This leads us to discuss the theories surrounding the treatment in FN.

### Treatment theories: (*information from textbook*)

The aim of FN treatment is to restore the optimal metabolism within the targeted neurons, i.e. the neurons constituting the “physiological lesion(s)”, in order to promote positive neuroplastic changes. By this process, normalization of their efferent outputs and thus a resolution (at least partial) of the patient’s symptoms is expected. This treatment is often multi-facetted and could include manual therapy but also the application of, for example, sensory, motor, or cognitive stimuli. Some such treatments were described in our four sources of information and were reported below in the section “Treatment modalities”.

Some rules are proposed to be followed for implementing a treatment in FN, specifically that: 1) the intensity of the stimulus has to be progressive and adapted to the degree of “fatigability” of the targeted group(s) of neurons; 2) the type of the stimuli and the side of their application depend on the characteristics of the stimulated pathways; 3) the stimuli have to be repeated and a single “physiological lesion” can be affected by several kinds of stimuli; and 4) the effects of treatment have to be assessed regularly by testing the positive indicators found during the initial clinical examination (e.g. assessing the decrease or the increase of “fatigability”) [39].

Concerning the intensity of the stimulus and the necessity of reassessing regularly the “fatigability” of the patient’s nervous system, in FN it is considered that neurons suffering from a dysfunctional CIS may be not able to support either an overly intense stimulation or too many repetitions of stimuli. The risk would be to aggravate the “physiological lesion(s)” [34]. This implies that parameters such as the “fatigability” of a group of neurons vary during treatment, appearing as a barometer of the treatment dose that the patient would be able to support.

In regards to the type and side of stimulation, these parameters refer to the fact that a variety of treatment modalities exist for acting on various parts of the nervous system. The choice of the type of stimuli depends on the targeted group(s) of neurons. The side on which they are delivered depends on whether the pathway that goes to the targeted neurons is crossed or uncrossed. In other words, a treatment modality is chosen for its expected ability to alter neuronal communication along a pathway until it reaches the “physiological lesion” of interest. For example, to reverse a “physiological lesion” of the left parietal cortex, the application of a source of vibration to joints of the right side of the body may be chosen [40]. Finally, the stimuli have to be repeated in the perspective of re-training the nervous system in order to cause lasting neuroplastic changes. The treatment is therefore dependent on the assumed area(s) of the defect nervous system and thus the same treatment can be provided for a multitude of diagnoses/symptoms.

### Clinical application of FN: (*information from all four sources*)

#### Indications: (*information from all four sources*)

All the groups of indications we identified were found in at least three of our four sources of information. Indications of FN are multiple with an emphasis on brain-related dysfunctions. Thus, according to our four sources, FN would be suitable to manage neuromusculoskeletal disorders, symptoms related to traumatic brain injuries, neurologic diseases or disorders, psychiatric disorders, and various neurologic or non-neurologic isolated symptoms. In addition, three sources showed that this approach would also be suitable for various conditions which did not fit with any of these groups of indications (see Table 2).

Among these indications, the following specific examples were reported by at least two sources: low back pain, neck pain, radiculopathies, peripheral neuropathies, upper and lower extremity conditions, Parkinson’s disease, multiple sclerosis, dystonias, migraines, complex regional pain syndrome, attention deficit disorder (ADD), attention deficit and hyperactivity disorder (ADHD), dyslexia, anxiety disorders, depression, post-traumatic stress disorders, obsessive compulsive disorders (OCD), balance disorders, and vertigo. Additional indications are listed in Table 2. In fact, several specific examples collected on the websites or through the interviews were apparently not reported in the literature.

#### Examination procedures: (*information from all four sources*)

As in other health disciplines, a detailed medical history is collected and the patient is observed, thus providing the first clues about which areas of the nervous system may present a physiological dysfunction. In addition, vital signs may be assessed and a general physical examination can be conducted. Complementary exams, e.g. magnetic resonance imaging and video-nystagmography, can also be recommended in order to detect/exclude a severe pathology and/or to supplement the functional neurological diagnosis.

However, the main aspect is the functional neurology examination. Table 3 provides a summary of diagnostic procedures used in FN according to our four sources. All of them reported the use of tests to assess the following: autonomic nervous system, sensory and motor components of spinal nerves, cranial nerves, reflexes, vestibulo-cerebellar system, cortical lobes and/or hemispheres, and cognition. The majority also reported assessing the basal ganglia. Specific tests are also mentioned, some of which are used to assess several structures or functions. For example, eye movements are used to assess the vestibulo-cerebellar system, the brainstem and/or the cerebral cortex, and finger-to-nose test is used for assessing the cerebellum and/or indirectly the cortical hemispheres.

**Table 3:** Diagnostic procedures used in Functional Neurology according to four sources in a scoping review

Structure(s) or function(s)		Sources of information			
		Book	Scientific articles	Websites	Interviews
Spinal nerve	Sensory	Spinothalamic tract^a^ Dorsal columns^b^	Spinothalamic tract^a^ Dorsal columns^b^	X	X
	Motor	Myotomes Muscle tone	Myotomes	Myotomes	X
	Reflexes	Osteotendinous Plantar Superficial abdominal	Osteotendinous Plantar	X	Osteotendinous
Cranial nerves		I to XII	At least, II to VIII, X to XII	At least, III, IV, VI, and VIII	I to XII
Vestibulo-cerebellar		Eye movements CN II, III, V, VII and, VIII to XII Romberg / Fukuda tests Finger-to-nose / Heel-to-shin tests Rapid alternative movements Vestibulo-ocular reflex Balance assessment Tandem gait Walking on toes / heels	Eye movements Finger-to-nose / Heel-to-shin tests Rapid alternative movements Vestibulo-ocular reflex Balance assessment Functional Romberg test	Eye movements Balance assessment	Eye movements Romberg / Fukuda tests Vestibulo-ocular reflex Balance assessment
Brain lobe(s)		Eye movements Blind spot mapping qEEG	Eye movements Blind spot mapping Gait assessment Finger dexterity Muscle testing Primitive reflexes Dual mental tasking	Eye movements Blind spot mapping	Eye movements Blind spot mapping
Basal ganglia		Looking for fascial tics	Colored lenses	NM	X
Autonomic		Observation (e.g. pupillary size, condition of the skin) Pupil light reflex Blood pressure Forehead / tympanic temperatures Heart rate Respiratory rate / ratio Oximetry Bowel auscultation Dermographia Vein-to-artery ratio of the retinal vessel	Blood pressure Heart rate Heart auscultation “Respiratory excursion” Vein-to-artery ratio of the retinal vessel Search for dermographia	X	Pupillary size or pupil light reflex Blood pressure Heart rate Oximetry
Cognitive		Questions about patient’s orientation and for testing memory	Wechsler intelligence scale for children Test of variables of attention Finger tapping test Cognitive tasks (e.g. memory tasks)	X	Test of variables of attention

*X* Structure(s) or function(s) mentioned without specific example(s)

*NM* Structure(s) or function(s) not mentioned in the source

*qEEG* Quantitative electroencephalography

^a^This includes nondiscriminative touch, temperature and pain sensations

^b^This includes fine touch, and conscious proprioception

Importantly, while most of the tests reported in Table 3 are commonly used in conventional neurological examination (e.g. myotomes and Romberg’s test) or in non-neurological examinations (e.g. vital signs), some of them are unusual or used differently in FN. The blind spot mapping is an example of such an unusual diagnostic test. The measurement of the vital signs to assess the CIS of the autonomic nervous system, which in turn is said to be able to reflect the CIS of cortical hemispheres, is an example of usual tests used and interpreted differently to what would usually be the case. This relationship between vital signs, autonomic nervous system and the CIS of the cortical hemispheres is said to be mediated by the cortico-reticulo-spinal tract (described in the section “Related neurological pathways”).

Tests may be used also without any obvious clinical indication and the interpretation of their results appears to be specific to FN, i.e. identification of one or more “physiological lesions”. The assessment of the cerebellar functions in a context of mechanical low back pain with spinal root compression illustrates the apparent “gap” between the clinical condition and the tests selected by the therapist [36]. In other words, to an “ordinary” clinician it would not be clear in which way tests of the cerebellar function would be relevant in mechanical low back pain.

It is our understanding that clinicians may take an individual approach to their diagnostic tests; either choosing specific tests based on the initial interview and observation of the patient or performing tests in order to screen for affected areas of the nervous system. It also appears that all tests are not used by all FN clinicians.

#### Treatment modalities: (*information from all four sources*)

Treatment modalities mentioned by our sources of information, as listed in Table 4, are coupled with the parts of the nervous system they are proposed to affect. As previously stated, treatment modalities appear to be primarily selected for their expected abilities to stimulate brain area(s) rather than in relation to the patient’s condition. The table shows how one brain area may be stimulated by several approaches and how one treatment modality may stimulate several areas. For example, eye movement exercises and manual therapy may be used to stimulate both the cortical hemispheres and the cerebellum. Another example is vibration therapy that may be used for these same areas, i.e. the cortical hemispheres and the cerebellum, as well as for the basal ganglia. In fact, the therapeutic modalities appear to include almost anything that would stimulate the nervous system, making it difficult to describe a treatment pattern. This treatment often includes home exercises to regularly stimulate the nervous system and it is often complemented with nutritional counseling or supplements. All the sources described the content of the treatment as individualized.

**Table 4:** Treatment modalities used in Functional Neurology according to four sources in a scoping review

Sources of information		Conditions or signs	Tissues at fault	Therapeutic modalities	Specific comments	General comments
Book	Chap.19	Migraines and vertigo	Right cerebral hemisphere	SMT Eye exercises Breathing exercises Nutritional therapy		Most of the treatment modalities (e.g. SMT, sound therapy, eye exercises) are provided or performed to the opposite side of the targeted hemisphere. Nutritional therapy consists mainly of vitamin B, omega 3 and C0Q10 supplementation.
		Complex regional pain syndrome	Cerebral hemisphere(s)	Joint manipulations Counting backwards Breathing exercises Nutritional therapy Hot and cold compresses Orthotics	The targeted hemisphere is probably the left because counting backwards is said by the author to stimulate the left cerebral hemisphere.	
		Attention deficit and hyperactivity disorder	Right cerebral hemisphere and left cerebellum	Joint manipulations Sound therapy Spatial rearrangement exercises Breathing exercises Nutritional therapy		
		Depression	Cerebral cortex	Joint manipulations Sound therapy Spatial rearrangement exercises Looking at old photos and making up stories about them Breathing exercises Nutritional therapy		
		Low back pain with spinal root compression	Right cerebral hemisphere	Joint manipulations Breathing exercises Nutritional therapy		
		Post SMT symptoms	Right cerebral hemisphere and left vestibulo-cerebellar system	Joint manipulations Soft tissue and trigger point therapy Breathing exercises Nutritional therapy		
	Chap.20	NA	Cerebral hemisphere	Activation: Any complex chore Manipulative therapy Eye exercises Cerebellar activation Sensory stimuli: visual, auditory, olfactory Transcutaneous electrical neural stimulation Inhibition: Earplugs, blinders Visualize rather than perform activities Evoked potentials at reduced amplitude	Some specific stimuli to stimulate the right and the left cerebral cortex are described. Moreover, some specific stimuli directed for the different lobes of the hemispheres are also described [39]. Stimuli directed to the cerebellum are described below.	In Chap. 20, the author does not deal with conditions but only with targeted neurological structures.
		NA	Cerebellum	Manipulative therapy Warming the auditory canal Revolving chair Eye movements Passive muscle stretch Squeezing a ball Pointing	Specific exercises to stimulate the medial part and the lateral part of the cerebellum are also proposed [39].	
		NA	Vestibule	Cawthorne-Cooksey exercises Balance exercises	For details concerning these exercises, see [39].	
		NA	Brainstem	Smell and/or taste food Exercises and/or stimuli of muscles innervated by cranial nerves Rectal dilation	Specific exercises to stimulate the mesencephalon are also mentioned [39].	
		NA	Sympathetic activity	Local application of warm Transcutaneous electrical neural stimulation	These modalities are described to inhibit the sympathetic activity.	
		NA	Caudate nucleus	Visualizing pleasant stimuli	In contrast, amygdala and/or hippocampus may be stimulated by visualizing unpleasant stimuli and “narrative recall” and list learning.	
Scientific articles	Carrick (1997) [9]	Enlarged physiological blind spot	Cerebral hemisphere	SMT		In the articles listed here, the large majority of the therapeutic modalities, i.e. manipulation, vibration therapy, eye exercises, and mirror therapy**,** are provided or performed depending on the targeted structure(s) and its/their side(s), except in the articles of Pedro (2005) (where this is not mentioned) and of Hirsh (2013) (where this is only mentioned for vibration therapy). ^b^These studies were conducted on healthy subjects who were found with an enlarged blind spot of one of their eyes.
	Pedro (2005) [41]	Landau-Kleffner syndrome	Left hemisphere and right cerebellum	Manipulation Eye movement exercises Visual, olfactory, auditory, vestibular and somatosensory stimuli Interactive metronome Nutrition therapy	There was no precision of which modalities would alter one of the two targeted structure rather than the other.	
	Daubeny (2010) [57]	Enlarged physiological blind spot	Cerebral hemisphere	Upper extremity manipulations		
	Bova (2013) [43]	Cervical dystonia	Left cerebral cortex (frontal lobe)	Eye movement exercises		
			Right cerebellum	SMT Vibration therapy		
			Right vestibular system	Eye movement exercises		
			Left basal ganglia	Eye movement exercises Vibration therapy Blue-lensed glasses		
	Kuhn (2013) [44]	Migraines, attention deficit and hyperactivity disorder, obsessive compulsive disorder, and Tourette’s syndrome	Right cortical hemisphere	SMT Coordination activities associated with eye movements Interactive metronome		
			Left cerebellum	SMT Coordination activities associated with eye movements Interactive metronome		
			Right basal ganglia	SMT Coordination activities associated with eye movements Interactive metronome		
			Left pons	SMT Coordination activities associated with eye movements		
	Hirsh (2013) [46]	Attention deficit and hyperactivity disorder, primary nocturnal enuresis and musculoskeletal pain	Right cortical hemisphere and left cerebellum	SMT Blue-lensed-glasses Optokinetic stimulation Vibration therapy Balance exercises Vestibular stimulation Timing exercises, including interactive metronome Home exercises: inhibitory of primitive reflexes, muscles strengthening, and balance exercises. Dietary changes	There was no precision of which modalities would alter one of the two targeted structure rather than the other.	
	Esposito (2013) [48]	Symptoms related to traumatic brain injury	Cortex (including frontal lobe)	Off-axis rotational device	Other modalities are used (see Additional file 2a) without clear mention of which neurological areas are targeted.	
			Vestibule	Off-axis rotational device		
			Right lower brainstem	Off-axis rotational device		
			Left upper brainstem	Off-axis rotational device		
			Superior colliculi	Red-blue-lenses		
	Bova (2014) [45]	Parkinson’s disease	Cerebral cortex	SMT Cross crawl exercises Mirror therapy	Cross crawl exercises are performed to stimulate the frontal lobe. Mesencephalon was also targeted without any mention of what modalities were used for.	
			Basal ganglia	Vibration therapy Blue-lensed glasses Mirror therapy		
	Bova (2014) [40]	Idiopathic hemiparesthesia	Left cerebral cortex (parietal lobe)	Vibration therapy	SMT and cold laser therapy were also used.	
			Left vestibular system	Eye exercises		
	Traster (2014) [47]	Symptoms related to traumatic brain injury	Left cerebral hemisphere	Manipulative therapy Passive complex movements of the extremities Eye movement therapies Earth-vertical axis rotations	Breathing exercises were also given to the patient.	
			Left brainstem (including the left superior colliculus)	Optokinetic stimulations		
			Overall vestibule	Eye movement therapies Earth-vertical axis rotations		
Websites	Website 4	Symptoms related to traumatic brain injuries	Vestibular system	Off-axis rotational device		The content of each treatment is individualized, following the statements of the five websites. All of the practitioners resort to eye exercises and to home exercises and/or lifestyle counseling, especially concerning nutrition (see Additional file 2b).
Interviews	Informant 1	NA	Temporal lobe(s)	Riding a bike		The content of each treatment is described as individualized. All the informants resort to home exercises. The majority of them use manipulative therapy and eye exercises (see Additional file 2c).
	Informant 2	NA	Cerebral hemisphere	Manipulative therapy		
		Symptoms following traumatic brain injuries	Brainstem	Somatosensory evoked potential		
	Informant 3	NA	Cerebral hemisphere	Manipulative therapy	Coordination exercises and exercises for fine motor skills are performed to stimulate the lateral part of the cerebellum.	
		NA	Cerebellum	Manipulative therapy Coordination exercises Exercises for fine motor skills		

*SMT* Spinal manual therapy

*NA* Not applicable

#### Treatment plans: (*information from scientific literature, websites and interviews*)

Concerning the treatment plans, we analyzed information from three sources (the book was excluded). On this basis, it seems clear that treatment plans are individualized. During the initial treatment period, regardless the conditions discussed, several appointments per week or even per day were proposed to patients. The period during which these treatment sessions are planned is variable but typically extended two weeks. Moreover, the use of home exercises appears quite common in addition to treatment with the therapist. Very little information is given in regard to the long-term strategies of care that might be established. This information and some details related to the course of care (e.g. duration of treatment sessions or home exercises) are available in Table 5.

**Table 5:** Treatment plans used in Functional Neurology according to four sources in a scoping review

Sources of information		Condition(s)	Initial care	Maintenance care
Articles	Pedro (2005) [41]	Landau Kleffner syndrome	Daily visits, 4.5 h per week, for 12 weeks	
	Beck (2009) [42]	Complex regional pain syndrome	1 to 2 visits per week for 8 weeks, plus 1 visit each 2 week for 1 month, plus home exercises	
	Kuhn (2013) [44]	Migraines, ADHD, OCD and, Tourette’s syndrome	42 visits over 19 weeks	
	Hirsh (2013) [46]	ADHD, primary nocturnal enuresis, and musculoskeletal pain	36 visits over 18 weeks, plus daily home exercises	
	Bova (2014) [45]	Parkinson’s disease	2 visits per week for 2 months, plus home exercises	After the initial care (i.e. 2 months), the frequency of 2 visits per week was maintained (for at least 8 months).
	Bova (2014) [40]	Idiopathic hemiparesthesia	3 visits in 2 weeks	
	Traster (2014) [47]	Symptoms related to traumatic brain injury	Approximately 2 to 3 visits per week for 3 months	
Websites	Website 1	In general	Individualized Usually, several times per day with an average of 3 times of 1.5 h each, for 1 to 2 weeks	
	Website 4	In general	Individualized Usually, 2 times per week for 6 weeks, plus home exercises	
		Complex conditions (type of conditions was not specified)	3 to 5 times per day for up to 5 consecutive days	
	Website 5	In general	Individualized Usually, 1 to 3 times per week for few weeks, plus home exercises This frequency is usually decreased over 2 to 4 months	Patient is often requested to do home exercises.
		Complex conditions (e.g. severe brain injuries, and advanced degenerative diseases)	Several visits per day for 1 to 2 weeks	
Interviews	Informant 1	In general	Individualized Usually 2 to 3 visits close in time, plus home exercises If good results are obtained, treatment is continued, more spaced in time. Daily visits or, 2 to 3 visits per week, may be needed, for 2 to 3 weeks.	
		Complex conditions (unspecified)		
	Informant 2	Moderate neurodevelopmental disorders	Individualized Usually, 1 to 2 visits per week for a few weeks, plus daily home exercises for about 10 min per day This frequency is usually progressively decreased	
		Severe neurodevelopmental disorders	Visits are more frequent than for the moderate form.	
	Informant 4	In general	Individualized Usually, 3 to 4 times (about 20 min each) per day for 2 to 3 weeks or 2 times per week for 3 to 4 months	
		Parkinson’s disease	Several visits per day for 3 consecutive days for 1 week	Patient is seen 3 to 4 times per year for the same treatment plan.

*ADHD* Attention deficit and hyperactivity disorder

*OCD* Obsessive compulsive disorder

#### Clinical outcomes: (*information from all four sources*)

Finally, we were interested in the factual or expected clinical outcomes. This is reported in the order of the scientific “credibility” of the sources. In general, websites and informants reported for various conditions, relief or recovery, but most of the time without mentioning the usual time course of recovery/improvement (see Table 6).

**Table 6:** Clinical outcomes reported and/or expected after treatment with Functional Neurological according to four sources in a scoping review

Sources of information		Conditions	Early clinical outcomes	Clinical outcomes with unspecified time frame	Long-term clinical outcomes
Book	Chapter 19 p.332–341	Complex regional pain syndrome	At 12 weeks, full recovery of function, persistence of bouts of pain		
		Migraines and vertigo	Less frequent migraines, resolution of vertigo		
		ADHD	At 12 weeks, improvement of concentration, reading ability and other academic abilities	*Further improvement is expected.*	
		Depression	At 12 weeks, improvement of depressive state	*Further improvement is expected with continued treatment.*	
		Low back pain with spinal root compression	At 12 weeks, pain free, but persistent episodes of numbness		
		Post manipulative therapy symptoms	At 12 weeks, resolution of imbalance and headaches, reduction of the other symptoms including confusion	*Further improvement or even resolution is expected with continued treatment.*	
Articles	Pedro (2005) [41]	Landau-Kleffner syndrome (case report)	At 12 weeks, improvement of language, auditory and motor skills		
	Beck (2009) [42]	Complex regional pain syndrome (case report)	At 12 weeks, full recovery of function, but persistence of bouts of pain		At 1 year, functional recovery is maintained, episodes of pain are reported.
	Bova (2013) [43]	Cervical dystonia (case report)		Functional improvement, decrease of spasmodic torticollis	
	Kuhn (2013) [44]	Migraines, ADHD, OCD, Tourette’s syndrome (case report)	At 19 weeks, migraines were gone, tics and, learning and behavioral capacities were improved		
	Hirsh (2013) [46]	ADHD, primary nocturnal enuresis, and musculoskeletal pain (case report)	At 18 weeks, improvement of behavior, confidence, and posture No more difficulty in daytime urinary control		At 3 months, occasional bed wetting and improvements in various activities of daily living
	Esposito (2013) [48]	Symptoms related to traumatic brain injury (case report)	At 10 weeks, improvement of balance, cognitive abilities, mood, and anxiety Decrease of the number and severity of physical complaints		
	Bova (2014) [45]	Parkinson’s disease (case report)	At 2 months, improvement of posture, function and well-being		At 10 months, treatment is continued twice per week for maintenance care with stable results. NB: Relapse was observed when treatment was reduced to once per week.
	Bova (2014) [40]	Idiopathic paresthesia (case report)	At 2 weeks, symptom free after 2 visits		
	Traster (2014) [47]	Symptoms related to traumatic brain injury (case report)	At 3 months, recovery of vibration sense, free of dysesthesia, and improvement of balance and gait		
Websites	Website 1	Unspecified		*Relief or resolution of patient’s symptom(s)*	
		Symptoms related to traumatic brain injury		*Resolution*	
		Parkinson’s disease, Alzheimer’s disease, and ADD / ADHD		*Improvement*	
	Website 2	Unspecified		*Relief of patient’s symptom(s)*	
	Website 3	Unspecified		*Significant relief or resolution of patient’s symptom(s)*	
		Migraines, and Post-concussion symptoms		*Resolution*	
	Website 4	Unspecified		*Relief or resolution of patient’s symptom(s)*	
	Website 5	Unspecified		*Profound relief or resolution of patient’s symptom(s)*	
Interviews	Informant 1	Most of the conditions	*Improvements of patient’s symptom(s), usually after 2 to 3 visits*		
		Vertigo	*“Good”, usually after 2 to 3 visits*		
		Tinnitus	*Less constant, usually after 2 to 3 visits*		
	Informant 2	Most of the conditions	*“Good”, usually after 2 to 3 weeks of treatment*		
		Neurodegenerative diseases, tremor disorders, high “fatigability” of the nervous system		*Less constant and longer to observe*	
	Informant 3	Reversible or “functional” conditions (e.g. vertigo, balance issues, headaches)	“Good”, and *potentially stable*, after 3 to 4 visits		
		Irreversible conditions		Results concerning some of the symptom(s) of the patient’s pathology, take longer to achieve, and *stable with maintenance care.*	
	Informant 4	Most of the conditions		*Improvement, usually transitory, of some of the patient’s symptom(s)*	
		Any pediatric conditions (e.g. ADD, cerebral palsy), and post-stroke symptoms, and chronic musculoskeletal disorders		Results are better than those described for the other conditions in adults. For children, results are also more stable.	

Expected clinical outcomes are reported in italic

*ADD/ADHD* Attention deficit disorder/Attention deficit and hyperactivity disorder

*OCD* Obsessive compulsive disorders

The textbook [36] reported on six different cases: i) complex regional pain syndrome, ii) migraines associated with vertigo, iii) ADHD, iv) depression, v) low back pain with spinal root compression, and vi) symptoms related to treatment by spinal manipulation. For these patients, clinical outcomes were reported as positive in general after twelve weeks, whether partial or complete. No clinical outcomes were reported beyond this period of treatment.

We found seven case studies in the literature reporting on: i) Landau-Kleffner syndrome [41], ii) complex regional pain syndrome [42], iii) cervical dystonia [43], iv) migraines, ADHD, OCD and Tourette’s syndrome [44], v) Parkinson’s disease [45], vi) idiopathic hemi-paresthesia [40], and vii) ADHD, primary nocturnal enuresis and musculoskeletal pain [46]. Two case studies were found that reported on symptoms post-traumatic brain injury [47, 48]. For these case reports, clinical outcomes were reported at various time intervals as positive, whether partial [41–48] or complete [40, 42, 44, 47]. Two case studies [42, 45] reported long term clinical outcomes, both describing patients as improved. One case study [46] reports the outcomes three months after cessation of care, describing the patient as being improved. No randomized controlled trials were found that could confirm the therapeutic effect of FN approach as a supplement to “traditional” chiropractic on any clinical outcome (for more information see Table 6). In fact, to the authors’ knowledge, no study design other than case-reports currently exist that describe therapeutic outcome in symptomatic patients.

## Discussion

### Brief summary of findings

To our knowledge, this is the first article to provide an overview of the theoretical framework and the clinical applications of FN, in the context of chiropractic manual therapy. In short, FN is described as a therapeutic approach that could be used for a large array of conditions, provided that the cause of such conditions can be traced primarily to parts of the central nervous system. The diagnosis is performed through the use of many conventional, but also more unusual tests, with a very “fine-tuned” interpretation of test results. In some cases, the fine tuning consists of looking for minor asymmetry and “subclinical lesions”. Treatment consists of various activities or therapies that are thought to affect clusters of neurons that have been diagnosed as dysfunctional. The initial treatment plan appears intense with several sessions per week or even per day. After this initial intervention period, it seems that the clinical outcomes are generally reported as positive, whether partial or complete, regardless of the condition of the patient (e.g. Parkinson’s disease, low back pain with radiculopathy).

### Methodological considerations

This information was obtained through a scoping review that included four sources: i) one textbook of FN, ii) eleven articles from the scientific literature, iii) the websites of five chiropractors proficient in FN, and iv) a semi-structured interview of four chiropractors who practice FN daily. As our research purpose was broad and FN is not well documented in the scientific or academic literature, we performed a scoping review rather than a number of rigorous systematic reviews, using for this multiple sources of information.

Our four sources helped us cover our six research objectives. However, the book was the only material that we used for the theoretical background of FN as it extensively informed us on its fundamental concepts. Few scientific articles were found in relation to our areas of inquiry; most of them case studies. Thus, websites of FN practitioners and the interviews were needed to collect enough information to make it possible to provide a clear and consistent picture of what constitutes FN. The latter two sources, i.e. websites and interviews, were also selected to fit the recommendations for conducting scoping studies [27, 28].

The representativeness of our sources of information and the validity of the extruded information seem to be satisfactory, as discussed below. Perhaps other researchers using alternative sources of information may have obtained varying results but it is our opinion that this review has reasonably captured the spirit and nature of FN, as there was good agreement between the various sources.

We decided to restrict the present review to FN theories and their clinical applications in the chiropractic context, i.e. we were interested only in sources that included the use of manual therapy. Thus, the work presented here does not depict the whole field of FN, which is wide and merits further explorations. As reported in the introduction, FN is currently composed of different sub-specialties which represent various forms of FN practice. These do not always include manual therapy but choose other therapeutic strategies, for example eye movement training [49–51], “hemisphere specific remediation programs” [52, 53], and music therapy [54].

i) **Textbook**
  Only the first author read the entire book and collected the relevant information for our work, which may be a methodological weakness. However, the understanding of the theoretical frame of FN was corroborated by the semi-structured interviews that contained several questions about fundamental concepts of FN, thus aiding in understanding. Further, the FN theories reported here, seem to be in agreement with how FN is defined by statements produced by some FN associations [23, 55]. Concerning our research objectives relating to clinical applications of FN, the information was straightforward to find because the textbook is well structured with specific chapters dedicated to those topics of interest to this work. Still, it is possible that some information may have been missed or misinterpreted.
ii) **Scientific literature**
  The usual strategy of searching for relevant articles by key words could not be used as it resulted in only one relevant article. The reason for this is that the term “functional neurology” is not usually used in such publications, maybe because FN covers many fields of clinical applications. Therefore, relevant keywords could not be predicted.
  Instead, we attempted a search by author, including at first all authors that appeared to publish in this area. However, this produced many irrelevant authors with the same surname and initials. Therefore, we stopped this strategy after the search for FR Carrick, RW Beck, G Leisman and R Melillo in the three selected databases. These four were selected because they appeared to be central to the FN movement. Thereafter, on the advice of a specialist librarian, we wrote directly to these four authors and authors known to have published with them, asking them for their lists of publications. In this way, five publication lists were obtained which resulted in no additional peer-reviewed articles being found. One of these authors recommended a search of the journal *Functional Neurology, Rehabilitation, and Ergonomics*. Three additional articles were found in this journal. Finally, our citation search did not result in any additional publications. The obstacles encountered in searching the literature made it difficult to appreciate if all relevant peer-reviewed articles were captured. However, all the acquired literature had the hallmarks of FN, as we had interpreted it from the other sources, so it is our impression that we managed to catch the essence of FN.
  The selection of articles for this review was made independently by the first author and an experienced researcher in the team. There were no disagreements between them. Further, the search for relevant information was also blinded with total agreement between the two.
iii) **Websites**
  The first author read the websites and collected the relevant information. These were read twice and blinded to previous findings. Subsequent readings would sometimes result in more information being included but no obvious misunderstandings appeared in the later readings.
iv) **Interviews**
  Each interview was conducted by the first author and another PhD student. Each tabulated transcription was done independently by these two people, with the option to listen again to the taped interview. This was necessary only once to clarify the content of the reply of one of the four informants to one of the 24 questions. No other differences between the content of these transcriptions were found between the interviewers.

### Synthesis of findings

According to our review, FN has a well described rationale that, if correct, has the potential of improving the lives for many people with a wide variety of conditions which are most often chronic and difficult to manage. The diagnosis of the neurological lesions in FN, i.e. the “physiological lesions”, certainly requires a solid background in central neurology. For chiropractors who embrace this approach, clinical practice would surely be both interesting and challenging.

Further, the practice of FN demands an understanding of how to test the various potential lesions. The interpretation of these tests seems to be very specific to FN, requiring them to be done bilaterally, attempting to detect asymmetries which would indicate lesions, also at a “subclinical” stage. In addition, the patient examination appears time consuming given the numerous tests that are performed, even when there is no obvious indication for them to be performed. Since these tests are used not only to detect lesions but also to monitor progress, the whole treatment strategy seems to be based on these tests; they have great importance, perhaps more than the symptoms. For these reasons, it would be relevant for FN users to assure that all their diagnostic tools are reliable and valid.

Since the recommended treatments do not appear to be noxious, even prolonged treatments are unlikely to cause any direct physical harm. Notwithstanding the approach being low risk, there are two other important aspects which need consideration. Firstly, the choice of one type of therapy may keep patients away from a possibly more suitable treatment; hence the need for comparative studies to determine relative efficacy. Secondly, frequent treatments during a long period of time are costly, potentially both to individuals and society, hence the need to show that they provide better results than less costly alternatives.

We noticed that the list of conditions amenable to improvement with FN is large and the conditions vary in type. However, only few of these have been described in the scientific literature and there seems to be a lack of studies on the effectiveness of the treatment. In relation to treatment effect, it may be difficult to conduct randomized controlled clinical trials on a treatment that concentrates on the underlying lesion(s) rather than on groups of patients with similar symptoms, because it might be difficult to find enough patients with sufficiently similar lesions to satisfy the methodological requirements for such studies. Obviously, case reports are not sufficient to “prove” the benefit of a treatment, unless the condition is truly irreversible and the observations absolutely objective and irrefutable, as many factors other than the treatment can make a patient feel or appear improved.

At early follow-up, the clinical outcomes in FN are generally reported as complete or partial, without a specific pattern related to the type of condition and/or the severity of the underlying neurological abnormalities. However, we could not judge the long-term clinical outcomes or prognosis for various conditions or in relation to the diagnosed neurological status from a lack of information in our sources. Such outcomes require documentation, to ensure that early improvements endure well past the initial placebo (honeymoon) effect.

Our final impression of FN is that it can be described as a complex alternative to the old variant of the chiropractic subluxation model [56], in which the chiropractor does not consider symptoms, but instead claims to treat the underlying “cause”. Furthermore, when this “cause” has been removed, symptoms will diminish or disappear. According to this traditional chiropractic concept, the “cause” is the vertebral subluxation.

Likewise with FN, the chiropractor does not deal directly with the presenting complaint, but is claiming to treat the underlying “cause”. The main differences are: i) that the “cause” is not as “simple” as the vertebral subluxation but one or more complex dysfunction(s) of the nervous system (often located in the brain), and ii) that the treatment is not limited to the spine and can be quite complex. In sum, the old variant of the chiropractic subluxation concept is spine-centered whereas FN embraces the whole nervous system, with an emphasis on the brain.

Verification of the scientific rationale of the theories of FN, evaluation of the validity of its treatment procedures, and consideration of the effectiveness of its treatments were beyond the remit of this scoping review. However, given that FN has been subject to lively criticism [10, 14–16, 20, 21] and the apparent paucity of scientific documentation within the domains we searched, it would be appropriate to scrutinize these aspects in future studies. This requirement would be the same for any therapeutic approach that is not an accepted part of mainstream medicine.

The neurological concepts presented by functional neurologists are varied and difficult to verify without having access to experts within many fields, willing to submerge themselves in this topic. A study of the plausibility of the concepts used in FN therefore would appear to be very difficult and time-consuming. Nevertheless, such studies may be justified but only if the treatment approach was tested and found to be valid.

A first step towards a validation of FN would therefore be to study whether one or several of the therapeutic tools suggested by the functional neurologists actually has an objective effect on the nervous system. If so, it would also be needed to investigate if this effect is clinically relevant and sustainable. For example, one central argument in FN is that joint manipulation has a powerful effect on the brain [9, 39, 57]. As some research has been conducted in this area [58–61], a review of the literature seems timely.

Another necessary, perhaps more simple, approach would be to test the validity of the clinical tests. Obviously, the diagnostic procedure has to be reproducible for the diagnosis to be valid. In turn, it is crucial to ensure that the treatment effect (if there is one) can be attributed to the purported mechanisms.

## Conclusion

The FN concept that reversible lesions in well-defined areas of the nervous system, especially of the brain, can be an identifiable cause of a multitude of disorders, is difficult for clinicians untrained in FN to verify. Nevertheless, the potential ability to change the quality of life for people suffering from poorly understood and/or chronic disorders makes this concept attractive for both clinicians and patients.

However, there is a need for more transparent documentation on the validity of the various steps normally considered important in evidence-based practice. In other words, the scientific community is waiting with interest to learn more about: i) the plausibility of the rationale of the various more unusual concepts of FN, ii) the reliability of its clinical tests and neurological diagnoses, and iii) the effect of treatment, particularly in relation to spinal manipulation, whether applied to musculoskeletal complaints or not.

## Additional files

Additional file 1: Appendices 1 Search strategy for scientific literature. **2.** Questions at a semi-structured interview on the use of Functional Neurology. (ZIP 30 kb)

Additional file 2: Appendices 3a Description of 11 peer-reviewed articles on Functional Neurology included in a scoping review. **3b.** Clinical information from websites of chiropractors using Functional Neurology. **3c.** Clinical information on the use of Functional Neurology (FN) from semi-structured interviews of chiropractors proficient in its use [62–66]. (ZIP 78 kb)

Additional file 3: Appendix 4. Data analysis and synthesis. (DOCX 109 kb)

## Abbreviations

ADD: Attention deficit disorder
ADHD: Attention deficit and hyperactivity disorder
CIS: Central integrative state
DACNB: Diplomate of the American Chiropractic Neurology Board
FN: Functional neurology
OCD: Obsessive compulsive disorder
PMRF: Pontomedullary reticular formation
