# Supplementary material for: Unravelling functional neurology: a scoping review of theories and clinical applications in a context of chiropractic manual therapy
Source: Chiropr Man Therap. 2017 Jul 18;25:19. doi: 10.1186/s12998-017-0151-1 (PMC5517058; doi:10.1186/s12998-017-0151-1)
Supplement: Supplementary file 1 — Search strategy for scientific literature. 2. Questions at a semi-structured interview on the use of Functional Neurology. (ZIP 30 kb) [file 12998_2017_151_MOESM1_ESM.zip › Additional file 1/App 1 Number of ref updated.docx]

**Appendix 1**: Search strategy for the scientific literature.

A preliminary search was done using the following search terms: « functional neurology », « chiropractic neurology » and « functional neurology AND chiropractic » in PubMed, PsycINFO and SPORTDiscus. We used no restrictions for date of publication. The search was restricted to English or French languages. However, we realized that scientific literature in this field could not be found in this way. Indeed, only one publication was found using the search terms « chiropractic neurology » [43].

We therefore changed our strategy, instead searching for authors known to publish within FN, i.e. initially FR Carrick and RW Beck, because they appear to be two of the main authors in FN. FR Carrick is considered as the founder of FN, and RW Beck is the author of, to our knowledge, the only FN textbook which deals with the topic in a context of manual therapy. Two frequent co-authors of FR Carrick, also known for their works in a specific branch of FN, i.e. FN applied to neurodevelopmental disorders, were then targeted in further searches, also by name of author (G Leisman and R Melillo). This search was completed in June 2016 and updated in November 2016. Although we found some relevant publications, the number of publications from authors with the same name who were not one of these four appeared often as other authors may be included in the databases in a same or similar manner.

As we had planned to extend our search to the co-authors of the publications we had found, a librarian from the University of Southern Denmark was consulted in order to 1) confirm that no additional publications could be found through search by keywords, and 2) attempt to resolve the problem in relation to the search by authors. The librarian confirmed that the results from the search by keywords was non-productive, even when trying to go further with additional MeSH terms. Concerning the search by authors, the librarian advised us instead to contact the authors directly asking them for their updated publication list to avoid the problem in relation to the indexation of the authors.

Therefore, we attempted to contact the four first authors and their twenty-four co-authors (who were identified at the later step of full text screening) by email. We also attempted to contact the two authors of the article found by keywords, i.e. J Bova and A Sergent. For six of these 30 authors, we did not find any contact information (professional email, professional websites, ResearchGate contact, or Linkedin contact) but the other twenty-two could be contacted. Seven authors replied to our email but only five of them forwarded an updated list of publications as requested or indicated where to find their publications. Furthermore, one of them recommended us to search in the journal *Functional Neurology, Rehabilitation, and Ergonomics,* affiliated to the *International Association of Functional Neurology and Rehabilitation*.

The search for authors had the advantage that it actually provided us with a series of articles, the reference lists of which were also scrutinized for suitable publications.
