# Supplementary material for: Unravelling functional neurology: a scoping review of theories and clinical applications in a context of chiropractic manual therapy
Source: Chiropr Man Therap. 2017 Jul 18;25:19. doi: 10.1186/s12998-017-0151-1 (PMC5517058; doi:10.1186/s12998-017-0151-1)
Supplement: Supplementary file 1 — Search strategy for scientific literature. 2. Questions at a semi-structured interview on the use of Functional Neurology. (ZIP 30 kb) [file 12998_2017_151_MOESM1_ESM.zip › Additional file 1/Appendix 2.docx]

**Appendix 2**: Questions at a semi-structured interview on the use of Functional Neurology.

Which are your indications for a Functional Neurology (FN) approach?

Which are the indications for which you have the best treatment results?

What is your diagnostic procedure in FN for these patients?

Do you have one general FN standard diagnostic procedure used in all your patients? If not, do you have different diagnostic routines for different types of conditions?

More generally, what are the FN diagnostic tests that you most often use?

What is a FN treatment and what are your goals when using FN?

What are the FN therapeutic modalities that you usually or most often use?

Could you give us an idea of how a FN treatment plan is conducted?

Do the results usually occur quickly and are they long-lasting?

Within FN, manipulation seems to be provided following rules of side and force, are they rules that you follow?

What is the effect of manipulation that you want to achieve, is it segmental and/or supra-segmental?
