# Supplementary material for: Unravelling functional neurology: a scoping review of theories and clinical applications in a context of chiropractic manual therapy
Source: Chiropr Man Therap. 2017 Jul 18;25:19. doi: 10.1186/s12998-017-0151-1 (PMC5517058; doi:10.1186/s12998-017-0151-1)
Supplement: Supplementary file 3 — Data analysis and synthesis. (DOCX 109 kb) [file 12998_2017_151_MOESM3_ESM.docx]

**Appendix 4**: Data analysis and synthesis.

**Objective 1** (Theories which constitute the basis of Functional Neurology):

As shown in **Table 1**, the **theories** on which Functional Neurology (FN) are based were exclusively extracted from the book [29] and not from our other sources. The reasons are that the scientific articles and websites were not expected to provide substantial information about this topic or only on scattered theoretical point(s), and that the interviews were designed mainly in order to understand the clinical applications of FN. On this basis, we attempted to provide a brief description of the fundamental concepts of FN, in the context of manual therapies.

**Objective 2** (Indications of FN):

The **indications** of FN were listed from our four sources in **Table 2**, which was constructed as follows: After identifying groups and subgroups of indications, specific examples were collected from our four sources and were listed to show if each source mentioned them or not. For some indications, examples were not needed (e.g. symptoms related to traumatic brain injuries), in such cases their mention was identified with a cross. While all the examples mentioned in the book and in the scientific articles were listed, the examples from the websites and from the interviews were listed only when at least two websites or two informants evoked the same indication. This approach was chosen due to the extensiveness of the indications collected through these two sources. However, all the indications mentioned are available in **Appendices 3b and 3c**. Given the research objectives of the controlled trial and of the randomized controlled trial, conducted on healthy subjects with an enlarged blind spot, these two studies did not provide information about indications of FN.

**Objective 3** (Diagnostic procedures of FN):

**Diagnostic procedures** were identified from our sources and were classified according to the structure(s) or the function(s) they are stated to assess. **Table 3** lists specific examples of diagnostic procedures for each. Only the diagnostic procedures that could clearly be identified were taken into account. For example, a procedure such as “assessment of neurological endurance” was not considered because of the impossibility to identify to what it refers. As seen in **Table 2**, for the websites and the interviews, examples were reported when at least two websites or two informants mentioned them. If specific examples for structure or function tests were not provided by at least two sources (for websites, and informants), this was indicated with a cross. All the examination procedures that were identified from these two sources are available in **Appendices 3b and 3c**.

**Objective 4** (Therapeutic modalities used in FN):

Given the large number of **therapeutic modalities** that are used in FN and the fact that they are clearly chosen in relation to the area(s) of the nervous system they are stated to stimulate, we have chosen to present them in a separate table (**Table 4**) in the following way: For each source, we searched for treatment modalities that are clearly mentioned in order to stimulate one or several specific parts of the nervous system. Thus, not all the articles, websites and the interviews were used to collect this information. This strategy allowed us to illustrate how a neurological area may be stimulated rather than having a list of treatment modalities without any context. Furthermore, all the collected therapeutic modalities are listed in **Appendices 3a to 3c**.

**Objective 5** (Treatment plans in FN):

Concerning **treatment plans**, information was extracted from three sources, scientific articles, websites and interviews. In fact, at this step we already knew that despite a chapter dedicated to six clinical cases in the textbook, the treatment plans were not detailed. In **Table 5** we separated initial care from maintenance care. Only the articles, the websites, and the interviews, which mentioned the frequency of treatment sessions, were used to collect this information. Treatment plans were searched in general terms and for specific conditions. Given the research objectives of the controlled trial and of the randomized controlled trial, these two articles did not provide information about treatment plans in FN.

**Objective 6** (Reported or expected clinical outcomes after a FN approach):

Our last study objective was to describe the **reported or expected clinical outcomes** in response to FN care. The clinical outcomes were collected in **Table 6** and discussed in terms of early outcome/prognosis and long-term outcome/prognosis. Clinical outcomes were searched in our four sources in general terms (websites and interviews) and for specific disorders (textbook, scientific articles, websites and interviews), remembering that effect of treatment can be examined only in randomized controlled trials. As for objectives two and five, the controlled trial and of the randomized controlled trial did not provide information about clinical outcomes related to FN approach.
