# Supplementary material for: Unravelling functional neurology: a scoping review of theories and clinical applications in a context of chiropractic manual therapy
Source: Chiropr Man Therap. 2017 Jul 18;25:19. doi: 10.1186/s12998-017-0151-1 (PMC5517058; doi:10.1186/s12998-017-0151-1)
Supplement: Supplementary file 2 — Description of 11 peer-reviewed articles on Functional Neurology included in a scoping review. 3b. Clinical information from websites of chiropractors using Functional Neurology. 3c. Clinical information on the use of Functional Neurology (FN) from semi-structured interviews of chiropractors proficient in its use [62–66]. (ZIP 78 kb) [file 12998_2017_151_MOESM2_ESM.zip › Additional file 2/Appendix 3a No changes.docx]

**Appendix 3a**: Description of 11 peer-reviewed articles on Functional Neurology included in a scoping review.

| **1st author**  **Year**  **Design** | **Conditions or symptoms** | **Diagnostic procedures** | **Therapeutic modalities** | **Targeted structures** | **Treatment plan** | **Reported clinical outcomes** |
| --- | --- | --- | --- | --- | --- | --- |
| Carrick  1997  Controlled trial | NA (because experiment on asymptomatic persons) | Blind spot measurement | Upper cervical SMT | One cortical hemisphere | NA | NA |
| Pedro  2005  Case report | Landau-Kleffner syndrome | -Physical and neurological exams (ND)  -Video electro-nystagmography  -Ocular-motor function  -Metabolic test(s) (ND) | -Manipulation  -OPK, visual, vestibular, olfactory, auditory, and somatosensory stimulation  -Interactive metronome  -Nutritional therapy (ND) | Left cortical hemisphere  Right cerebellum | 4.5 hours per week, during 12 weeks | Improvement of language, auditory and motor skills after 12 weeks of treatment |
| Beck  2009  Case report | Complex regional pain syndrome type I | -Vital signs  -Auscultation of heart, lungs and abdomen  -Ophthalmoscopic and otoscopic exams  -Sensory examination  -Various reflexes  -Cranial nerves  -Eye movements  -Search for dermographia  -Ranges of motion  -Palpation | -Mobilizations and manipulations of the lower  extremities  -SMT  -Rehabilitation strategies  -Nutrition therapy  -Breathing exercises | Unspecified | 1 to 2 appointment(s) per week for 8 weeks plus 1 appointment every 2 weeks during 1 month | Full functional recovery after 3 weeks of treatment  Persistence of brief episodes of pain at 1 year |
| Daubeny  2010  Randomized controlled trial | NA (because experiment on asymptomatic persons) | Blind spot measurement | Manipulative therapy of the upper extremities | One cortical hemisphere | NA | NA |
| Bova  2013  Case report | Cervical dystonia (spasmodic torticollis) | -Observation  -Blind spot measurement  -Colored lenses  -Vibration therapy  -Eye movements  -Functional Romberg’s test | -SMT of the cervical spine  -Eye exercises  -OPK tape reflex therapy  -Vibration therapy  -Blue-lensed-glasses | Left cortical hemisphere (mainly frontal lobe) and left basal ganglia  Right cerebellum and right vestibular system | Unspecified | Functional improvement and decrease of spasmodic torticollis |
| Kuhn  2013  Case report | Migraines  Attention deficit and hyperactivity disorder  Obsessive compulsive disorder  Tourette syndrome | -Observation  -Dermatomes, myotomes, reflexes (upper and lower extremities)  -Cranial nerves, including light reflex  -Various cerebellar tests  -Eye movements  -Finger dexterity  -IM  -TOVA test  -Cornners’ Parent Rating Scale  -Orthopedic exams (ND)  -Palpation | -SMT of the cervical, lumbar and pelvic regions  -Eye exercises along with coordinated activities (ND)  -IM | Right cortical hemisphere and right basal ganglia  Left cerebellum and left pons | 42 visits over 19 weeks | Migraines were gone  Tics also, except in extreme situations of stress or emotion  Improvement in learning and behavioral capacities |
| Hirsh  2013  Case report | Attention deficit and hyperactivity disorder  Primary nocturnal enuresis  Musculoskeletal pain (cervical, back and ankles) | -Observation  -Balance assessment  -Blood pressure  -Heart rate  -Myotomes  -Rapid alternative movements  -Finger to nose test  -Primitive reflexes  -TOVA test  -Blind spot mapping | -SMT  -Blue-lensed-glasses  -OPK stimulation  -Vibration therapy  -Balance exercises  -Vestibular stimulation  -Timing exercises (including IM)  -Home exercises: inhibitory of primitive reflexes, muscles strengthening, and balance exercises.  -Dietary changes | Right cerebral hemisphere  Left cerebellum | 36 visits over a period of 18 weeks and daily home exercises | At 18 weeks:  Improvement of behavior, confidence, and posture  No more difficulty in daytime urinary control  3 months after cessation of care:  Occasional bed wetting and improvements in various activities of daily living (e.g. getting ready for school) |
| Esposito  2013  Case report | Symptoms related to traumatic brain injury (including: balance issues, dizziness, headaches with scintillating stocoma, anxiety, depression, cognitive impairments, and debilitating physical complaints) | -Observation  -Vital signs  -Ophthalmoscopic exam  -Pupil light reflex  -Balance assessment  -Cranial nerves  -Eye movements  -Sensory examination  -Reflexes  -Finger to nose/Heel to shin tests  -Red-blue-lensed-glasses  -Dual mental tasking  -Cognitive tasks  -Finger to thumb tapping test | -Manipulative therapy of cervical spine and of upper limb  -Eye movements  -Gyroscope  -Red-blue lenses  -Passive complex movements of the right upper and lower extremities | Cortices  Vestibule  Right lower brainstem  Left upper brainstem  Superior colliculi | 10 weeks of treatment (without more details) | Improvement of balance, cognitive abilities, mood, and anxiety  Decrease of the number and of the severity of physical complaints |
| Bova  2014  Case report | Parkinson’s disease | -Observation  -Eye movements  -Colored lenses  -Ranges of motion  -Finger-to-nose test | -SMT of thoracic spine  -Vibration therapy  -Blue-lensed-glasses  -Mirror imaging  -Cross crawl exercises | Frontal lobe  Basal ganglia  Mesencephalon | 2 visits per week for 10 months  The patient pursues this frequency of care during at least 10 months | Improved function, posture, tremor and well-being at 2 months  These results are stable at 10 month of follow-up.  A relapse was observed when the frequency of visits was decreased to 1 visit per week. |
| Bova  2014  Case report | Idiopathic hemiparesthesia | -Sensory examination  -Myotomes  -Reflexes  -Cranial nerves  -Vestibulo-cerebellar tests  -Eye movements  -Palpation  -Ranges of motion | -SMT  -Eye exercises  -Vibration therapy  -Cold laser therapy | Left parietal lobe  Left vestibule (posterior semicircular canal) | 3 treatment sessions in 2 weeks | Symptom free after 2 sessions of treatment |
| Traster  2014  Case report | Symptoms related to traumatic brain injury (loss of vibration sense in both legs, dysesthesia of plantar surfaces of the foot, balance and gait issues) | -Observation  -Auscultation of heart and lungs  - “Respiratory excursion”  -Ophthalmoscopic exam  -Cranial nerves  -Eye movements  -Sensory examination  -Myotomes  -Reflexes  -Plantar reflex  -Romberg test  -Finger-to-nose/Heel to shin tests  -Rapid alternative movements  -Finger tapping test  -Dual mental tasking  -Palpation | -Manipulative therapy  -Earth-vertical axis rotations  -Eye exercises, including OPK stimulations  -Passive complex movements of the right upper and lower extremities  -Breathing exercises | Left cortical hemisphere and left brainstem (including the left superior colliculus)  Overall vestibule | Approximately 2 to 3 times a week for 3 months | Recovery of vibration sense in legs and free of foot dysesthesia  Balance and gait improvement |

NA: Not applicable

SMT: Spinal manipulative therapy

ND: Not detailed in the article

OPK: Optokinetic

IM: Interactive metronome

TOVA: Test of variables of attention
