# Supplementary material for: Unravelling functional neurology: a scoping review of theories and clinical applications in a context of chiropractic manual therapy
Source: Chiropr Man Therap. 2017 Jul 18;25:19. doi: 10.1186/s12998-017-0151-1 (PMC5517058; doi:10.1186/s12998-017-0151-1)
Supplement: Supplementary file 2 — Description of 11 peer-reviewed articles on Functional Neurology included in a scoping review. 3b. Clinical information from websites of chiropractors using Functional Neurology. 3c. Clinical information on the use of Functional Neurology (FN) from semi-structured interviews of chiropractors proficient in its use [62–66]. (ZIP 78 kb) [file 12998_2017_151_MOESM2_ESM.zip › Additional file 2/Appendix 3b Number of refs updated.docx]

**Appendix 3b**: Clinical information from websites of chiropractors using Functional Neurology.

| **Reference number**  (in the order in which the websites were scrutinized) | [62] | [63] | [64] | [65] | [66] |
| --- | --- | --- | --- | --- | --- |
| **Professional profile of the leading practitioner** | Chiropractor DACBN  Practices in the US  Uses also “functional medicine” | Chiropractor, DACNB, FACFN  Practices in the US  Completed courses in clinical nutrition  Works with a nutritional therapist in his office | Chiropractor DACBN  Certified in FNOR  Practices in the US  Speaker in the Carrick Institute  Works with another chiropractor who is specialized in “functional medicine” | Chiropractor DACBN, FACFN, FABBIR  Formed in the FNOR approach  Practices in the US  Speaker in the Carrick Institute  Completed courses in “functional medicine” | Chiropractor DACBN, FACFN  Practices in the US  Speaker in the Carrick Institute  Gives lectures on “functional medicine” and nutritional approaches |
| **Mentioned indications for a functional neurology approach** | Parkinson / Alzheimer’s diseases  Dementia  Multiple sclerosis  Seizure disorders  Cerebral palsy  Cerebellar ataxia  Neurodevelopmental disorders, including dyslexia, autism, ADD, ADHD  PTSD  Tourette syndrome  OCD  Eating disorders  Anxiety  Headaches / Migraines  Cerebral vascular accident  Traumatic brain injury  Vertigo  Sleeping difficulties  Stress  Incomplete spinal cord injury  Limb amputation  Physical, academic, cognitive and artistic performances | Early Alzheimer’s / Parkinson’s symptoms  Dystonia  Tremor disorders  Fibromyalgia  Restless legs  Neurodevelopmental disorders, including ADD, ADHD, dyslexia and autism  Headaches / Migraines  Neck pain / Low back pain / Sciatica (e.g. bulging, herniated discs)  Spinal stenosis  Pain in upper and lower extremities  Carpal tunnel syndrome  Traumatic brain injury  Balance disorders  Vertigo / dizziness  Numbness  Insomnia | Early Alzheimer’s symptoms  Dystonia  Tremor disorders  Fibromyalgia  Restless legs  Headaches / Migraines  Neck pain / Low back pain / Sciatica (e.g. bulging, herniated discs)  Spinal stenosis  Pain in upper and lower extremities  Carpal tunnel syndrome  Traumatic brain injury  Balance disorders  Vertigo / dizziness  Numbness  Insomnia  Physical performances  Low immunity | Parkinson’s disease  Multiple sclerosis  Dystonia  Neurodevelopmental disorders, including ADD, ADHD and learning disabilities  Anxiety / Panic disorders  Depression  PTSD  Headaches / Migraines  Neck pain / Low back pain/Sciatica / Spinal stenosis  Pain in upper and lower extremities  Carpal tunnel syndrome  Temporomandibular joint disorders  Traumatic brain injury  Post stroke symptoms  Balance disorders  Vertigo / dizziness  Brain fog  Bed wetting  Spinal cord injury  Physical performances  Declining brain function related to aging  Lyme disease  Hashimoto’s disease  Andropause  Biotoxin illness  Food allergies or sensitivities  Leaky gut syndrome  Disembarkement syndrome | Parkinson / Alzheimer’s diseases  Multiple sclerosis  Dystonia  Tremors disorders  Seizures  Neurodevelopmental disorders, including dyslexia, autism, ADD / ADHD  Anxiety / Panic disorders  Depression  PTSD  Tourette syndrome  OCD  Headaches / Migraines  Chronic Pain  Peripheral neuropathies  Post-stroke symptoms  Traumatic brain injury  Balance disorders  Vertigo/dizziness  Fatigue  Sleeping difficulties  Physical, cognitive and creative faculties  Declining brain function related to aging  Lyme disease |
| **Examination procedures mentioned** | Detailed health history  Postural analysis  Cognitive tests  Autonomic functions  Ophthalmoscopic examination  Myotomes, dermatomes and reflexes  Examination of cranial nerves (at least some of them)  Balance assessment, including computerized dynamic posturography  Video nystagmography  Eye movements  Nutritional and metabolic screening  Interactive metronome  Quantitative electroencephalography | Not described | Health history  Postural and gait analysis  Cognitive tests  Myotomes and reflexes  Cerebellar tests  Balance assessment  Eye movements  Laboratory testing  Complementary imaging  Orthopedic assessment | Detailed health history  Postural and gait analysis  Cognitive tests  Vital signs  Balance assessment, including computerized dynamic posturography  Video nystagmography  Eye movements, including saccadometry  Coordination  Laboratory testing  Complementary imaging  Motor accuracy  Neurological endurance | Detailed health history  Postural and gait analysis  Examination of cranial nerves (at least some of them)  Cerebellar tests  Balance assessment, including assisted by computer  Video nystagmography  Eye movements  Laboratory testing |
| **Treatment modalities used within a Functional Neurology approach** | Individualised treatment that may include:  Musculoskeletal intervention  Motor exercises  Eye movements  Vestibular and balance training  Visual, auditory and olfactory stimulation  Computer based brain games  Interactive Metronome  Neurofeedback associated or not with transcranial magnetic stimulation  Metabolic and nutritional therapy  Oxygen therapy | Individualised treatment that may include:  Traditional chiropractic instruments and/or adjustments  Eye movements  Visual, auditory and olfactory stimulation  Heat  Changes of diet  Supplements  Lifestyle advice | Individualised treatment that may include:  Chiropractic adjustment  Soft tissues therapies  FNOR system, including rehab exercises  Eye movements  Vestibular rehabilitation  Repositioning maneuvers  Heat and ice application  Home exercises  Nutritional counseling  Lifestyle advice | Individualised treatment that may include:  Light force chiropractic adjustments  Soft tissues therapies, including nerve flossing exercises and ART technique  Rehab exercises  Eye movements  Balance rehabilitation  Vestibular rehabilitation, including an off-axis rotation device  Visual and auditory stimulation  Dynavision D2  Repetitive peripheral somatosensory stimulation  Accelerated recovery performance wave  Primitive reflex remediation  Home therapies  Changes of diet  Supplements | Individualised treatment that may include:  Chiropractic manipulation  Rehab exercises  Eye movements  Balance exercises  Vestibular exercises  Visual, auditory and vibration stimulation  Home exercises  Changes of diet  Supplements |
| **Mentioned treatment plans in relation to FN approach** | Individualised  Usually multiple sessions per day (with an average of 3 sessions of 1.5 hours each per day), for one or more weeks (e.g. during 5 to 15 days for enhancing academic performances)  No information on any long-term strategies | Not described | Not described | Individualised  Usually 2 times per week for 6 weeks plus additional treatments as needed  For complex conditions, the therapy may be more intensive: 3 to 5 times per day for up to 5 consecutive days  No information on any long-term strategies | Individualised  Usually 1 to 3 times per week for a few weeks and more frequently for challenging conditions (e.g. in severe brain trauma) with several sessions per day over one or two weeks.  Frequency of appointments decreases during a period of 2 to 4 months.  No information on any long-term strategies |
| **Expected clinical outcomes** | Relief / improvement (e.g. Parkinson’s disease, Alzheimer’s disease, ADHD) or resolution (e.g. post-concussion) of symptoms or conditions are expected.  Significant results in few days or weeks are expected.  The durability of the clinical outcomes is not mentioned. | Relief of symptoms or conditions is the main treatment objective  NB: FN treatment is described as not trying to cure anything.  Amazing results for symptoms and conditions that other methods fail to manage might be expected.  The durability of the clinical outcomes is not mentioned. | Significant improvement of symptoms or of conditions are expected.  Resolution of migraines, of vertigo, of chronic pain, and of symptoms related to brain injury could be expected.  The durability of the clinical outcomes is not mentioned. | Rapid decrease in pain and improvement in function are expected after the initial care.  Also, full recovery could be expected.  The durability of the clinical outcomes is not mentioned. | Profound improvement and even resolution of the symptoms, even for advanced conditions are expected.  NB: This statement is nuanced by specifying that FN does not cure or change degenerative conditions.  NB: At times, no results are observed.  The durability of the clinical outcomes is not mentioned.  NB: Often, home exercises are maintained beyond the supervised treatment in order to stabilize the results. |

DACNB: Diplomates of the American Chiropractic Neurology Board

US: United States

FACFN: Fellow of the American College of Functional Neurology

FNOR: Functional Neuro-Orthopedic Rehabilitation (for more information please see **http://fnor.net**)

FABBIR: Fellow of the American Board of Brain Injury and Rehabilitation

ADD: Attention deficit disorder

ADHD: Attention deficit and hyperactivity disorder

PTSD: Post traumatic syndrome disorder

OCD: Obsessive compulsive disorder

Dynavision D2 (for more information please see **http://www.dynavisioninternational.com**)

Accelerated recovery performance wave (for more information please see **http://www.arpwave.com**)
