# Supplementary material for: Unravelling functional neurology: a scoping review of theories and clinical applications in a context of chiropractic manual therapy
Source: Chiropr Man Therap. 2017 Jul 18;25:19. doi: 10.1186/s12998-017-0151-1 (PMC5517058; doi:10.1186/s12998-017-0151-1)
Supplement: Supplementary file 2 — Description of 11 peer-reviewed articles on Functional Neurology included in a scoping review. 3b. Clinical information from websites of chiropractors using Functional Neurology. 3c. Clinical information on the use of Functional Neurology (FN) from semi-structured interviews of chiropractors proficient in its use [62–66]. (ZIP 78 kb) [file 12998_2017_151_MOESM2_ESM.zip › Additional file 2/Appendix 3c.docx]

**Appendix 3c**: Clinical information on the use of Functional Neurology (FN) from semi-structured interviews of chiropractors proficient in its use.

| **Informant** (in the order in which the informants were interviewed) | **1** | **2** | **3** | **4** |
| --- | --- | --- | --- | --- |
| **Professional profile of the informant** | Informant is certified in FN (DACNB) and practices FN since more than 15 years  -Continues taking courses in FN  -Uses FN for 80% of his patients | Informant is certified in FN (DACNB) and practices FN since more than 20 years  -Continues taking courses in FN  -Uses FN for 100% of his patients | Informant is not certified in FN and, practices FN since more than 5 years  -Continues taking courses in FN  -Uses FN for 20 - 30% of his patients | Informant is certified in FN (DACNB) and, practices FN since more than 10 years  -Continues taking courses in FN  -Uses FN for 100% of his patients |
| **Typical indications for a FN approach** | All conditions can potentially be analyzed through FN.  **Examples of symptoms and conditions treated**:  -Multiple sclerosis  -Amyotrophic lateral sclerosis  -Post-concussion symptoms  -Musculoskeletal disorders (e.g. sciatica, sacroiliac joint pain)  -Vertigo  -Tinnitus  **Examples of other indications managed by other functional neurologists**:  -Post-traumatic stress disorders  -Medullary lesions | All conditions can potentially be analyzed through FN.  **Symptoms and conditions most often treated:**  -Neurodevelopmental disorders, including ADD/ADHD and « dys » disorders (e.g. dyslexia, dyspraxia)  -Migraines, headaches  -Post-concussion symptoms  -Vertigo, balance disorders  -Neuralgia (e.g. sciatica, Arnold neuralgia, trigeminal neuralgia)  **Other conditions also seen**:  -Neurodegenerative disorders (e.g. Parkinson's disease)  -Dystonia  -Musculoskeletal conditions (e.g. tendinopathies, low back pain) | **Symptoms and conditions most often treated**:  -Neuro-musculoskeletal conditions (recurrent or chronic)  -Migraines, headaches  -Vertigo, balance disorders  **Other conditions also seen**:  -Multiple sclerosis  -Post-stroke symptoms  -Neurodevelopmental disorders such as ADD/ADHD, and dyslexia | All conditions can potentially be analyzed through FN.  **Symptoms and conditions most often treated:**  -Parkinson’s syndromes  -Cervical dystonia  -Post-stroke symptoms  -Cerebral palsy  -Post-traumatic stress disorders  -Post-concussion symptoms  **Other conditions also seen**:  -Neurodevelopmental disorders (ADD/ADHD and « dys » disorders)  -Musculoskeletal disorders, especially the chronic ones (e.g. neck and low back pains, sciatica, headaches) |
| **Typical examination procedures for FN diagnosis** | The informant has a routine diagnosis which constitutes a basis on which some tests are added depending on the symptoms of the patient  **Diagnosis procedures**:  -Gait analysis (eyes open, eyes closed)  -Posture analysis  -Muscle testing of the proximal muscles and of the deep muscles of the neck  -Reflexes, including osteotendinous reflexes  -Brainstem evaluation, especially through the assessment of vison, vestibulo-ocular reflex and balance  -Balance analysis, including Fukuda test, force platform (eyes open, eyes closed, feet apart, feet together)  -Chiropractic palpation  **Other procedures regularly used**:  -Muscle testing of the distal muscles  -Blind spot mapping | Informant has no routine for patient examination which is rather individualized but uses a basis on which some other tests may be added  **Tests more often used**:  -Observation (e.g. looking for movement disorders, gait analysis, how the patient speaks)  -Blood pressure  -Oximetry  -Sensory examination, including pinwheel test and vibration sense  -Reflexes, particularly osteotendinous reflexes  -Examination of cranial nerves  -Vestibulo-cerebellar tests (e.g. Romberg, Fukuda, finger-to-nose/nose-to-finger, heel-to-knee, eyes movements)  -Eyes movements (pursuits, saccades, fixation and optokinetic movements), in part for assessing the function of the cerebral cortex  -Blind spot mapping  -Assessment of the function of the basal ganglia (e.g. finger tapping test)  **Tests less often used**:  -Cognitive tests (e.g. Test of variables of attention)  -Cardiac auscultation  -Cutaneous reflexes  -Balance platform  -Interactive metronome | **Diagnostic procedures:**  -Questionnaire about some functions of different parts of the brain  -Questionnaire about other systems than the nervous system (e.g. digestive, adrenal, bloodstream)  -Observation (posture and gait analysis, tonus of facial muscles)  Informant described the FN examination as individualized to be based on the replies to the questionnaires mentioned above.  **Tests more often used following the questionnaires**:  -Cerebellar tests  -Eyes movements (pursuits, saccades)  -Vestibulo-ocular tests  -Romberg test  -Fukuda test combined with a vestibular stimulation  -Pupillary reflex  **Tests previously used**:  -Blind spot mapping  -Posture analysis related to « hemisphericity » | **Diagnosis procedures**:  -Questionnaires, including one designed to detect an hemisphericity and one about nutrition  -Observation (looking for movement disorders, gait analysis, how the patient speaks)  -Cognitive tests  -Autonomic parameters, including oxygen, blood pressure, cardiac frequency and pupillary size.  -Peripheral neurological tests  -Examination of cranial nerves  -Static and dynamic vestibular tests (e.g. head impulse test)  -Cerebellar tests  -Eyes movements (including saccades, pursuit and optokinetic movements)  -Balance platform  -Feedback tests  -Somatosensory evoked potentials  Assessment of other parameters if necessary, depending on patient’s symptoms (e.g. the Test of variables of attention for children and adults who suffer from cognitive disturbances) |
| **Typical treatment modalities used** | All the chiropractic tools or techniques can be used within a FN approach.  Home exercises, including:  -Riding a bike (in order to stimulate the temporal lobes)  -Muscles strengthening (mainly, shoulder girdles, pelvic girdles and deep neck muscles)  Informant uses other treatment modalities such as ocular exercises for very specific problems. | First, uses chiropractic manipulation(s) and/or chiropractic tools  Thereafter, (depending on the case), treatment may consist on:  Transcutaneous electrical nerve stimulation  Somatosensory evoked potential, especially to stimulate the brainstem  Interactive metronome  Vibration therapy  Home exercises, including:  -Oculomotor exercises  -Vestibular exercises  -Physical exercises  Dietary advices (if needed) | First, uses chiropractic manipulations  Thereafter, (depending on the case), treatment may consist on:  Sensory stimuli, including stretching, visual, olfactory, and sound stimuli  Balance exercises  Oculomotor exercises  Coordination exercises  Exercises for fine motor skills  Home exercises  Mirror therapy  Digestive problems, body inflammation, disorders of blood glucose, etc. are aspects that have to be addressed (if needed) in order to facilitated the treatment by neuro-stimulations. | First, uses chiropractic manipulation and joint mobilization  Thereafter, (depending on the case), treatment may consist on:  Sensory stimuli, including vibration therapy, massage, sound, and light  Vestibular rehabilitation, including with the Gyrostim  Feedbacks, including neuro and myofeedback  Stimulation by sensory substitution  Oculomotor exercises  Somatosensory evoked potential  Transcutaneous electrical nerve stimulation |
| **Typical treatment plans** | Treatment plans are individualized, depending on the severity of the symptoms.  Usually, the informant plans 2-3 treatment sessions for observing if good results appear and then continue or not.  Severe conditions can need daily treatment or 2-3 sessions per week, during 2-3 weeks. However, the informant does almost not use this kind of intensive treatment plan due to the cost that would represent for the patients and the material that is needed. If these types of care are needed, the informant tries to work with other practitioners who will maintain the results (e.g. physiotherapist). | Treatment plans are individualized, depending on the « fatigability » of the patient’s nervous system and on his/her implication in the treatment.  For example, an adolescent with moderate « dys » symptoms may be seen 1-2 times per week during a few weeks, then visits are progressively spaced. In addition, patients are supposed to do 10 min of therapeutic exercises per day at home.  A child also suffering from « dys » disorders but with more complex test results during the FN examination will be seen more frequently. | Treatment plans are individualized, depending on the symptoms of the patient and on his/her implication in home exercises.  Usually, symptoms and conditions for which the use of FN is needed require more frequent treatment sessions than with a « classic » chiropractic approach.  Home exercises have to be repeated several times per day (7-9) for short periods of 3-4 minutes. | Treatment plans are individualized, depending to the symptoms on the patient and on the therapeutic objectives.  Usually, patients are treated 3-4 times per day during 2-3 weeks or 2 times per week during 3-4 months. Each treatment session lasts about 20 minutes.  Patients who suffer from Parkinson's disease, are usually treated during 3 consecutive days, 3-4 times per year. |
| **Expected outcomes following a FN approach** | Varies, depending on patients’ symptoms and condition  For example, the informer has good results on vertigo and less consistently good results for tinnitus.  Generally, the results can be observed quickly, in 2-3 treatment sessions.  Sometimes, in few seconds, the awakening of neural circuits might be observed.  For most of the conditions, the results with a FN approach are not more important or quickly observed than with other chiropractic approaches. However, for complex cases, such as multiple sclerosis or post-concussion symptoms, FN may provide better and quicker results.  The stability of the results is comparable to other therapeutic approaches. This depends on the lifestyle of the patient. | Varies, depending on patients’ symptoms and condition  For most conditions treated by this chiropractor (e.g. headaches, vertigo, ADHD), good results are expected and they can be observed quickly, in 2-3 weeks of treatment.  Generally, some positive results are expected within 2-3 weeks. It takes longer to stabilize the condition.  The results take longer to occur for patients with a high degree of cellular « fatigability » and for neurodegenerative diseases. Results are less constant for neurodegenerative diseases and for movement disorders.  Treatment plan, treatment results and stability are influenced by the assiduity of the patient.  Results are described as longer lasting and “deeper” than with « classic » chiropractic. | Varies, depending on patients’ symptoms and condition  Usually, for headaches, vertigo and balance disorders (qualified as “functional symptoms” by the informant), good and quick results (in 3 or 4 treatments) are expected.  The results take longer to arrive, are more progressive and less impressive for irreversible conditions such as Parkinson’s disease or Alzheimer’s disease.  The results depend also on the assiduity of the patient with his/her home exercises and/or of his/her assiduity to return for maintenance care.  Stability of the results depends on the same criteria as those influencing the expected outcomes. The more the problem is chronic and/or “pathologic” vs. “functional”, the more chiropractic treatments can be needed to maintain the results. | The best results are noted for children in general (e.g. ADD, cerebral palsy) and, for patients suffering from chronic musculoskeletal disorders or from post-stroke symptoms.  For most conditions treated by this chiropractor, the results can be observed quickly. For example, a child who suffers from cerebral palsy can be improved after 3 days of treatment.  Sometimes the results are immediate. However, because of the severity of the encountered conditions, « miracles » are not expected.  Therapeutic objectives are defined with the patient. Generally, they aim for more autonomy.  Some patients do not respond to a FN treatment or only obtain minor improvement.  Usually, the results are transitory in adults and aging patients and lasting in children, even when they suffer from a severe condition. Also, it depends on the pathology and on the plasticity of the individual’s nervous system. |

DACNB: Diplomates of the American Chiropractic Neurology Board

ADD/ADHD: Attention deficit disorders / attention deficit and hyperactivity disorders
